# Supplementary material for: Configuration analysis of crop-pollination service management: a novel insight from the theory of planned behavior
Source: PLoS One. 2025 Jul 11;20(7):e0326226. doi: 10.1371/journal.pone.0326226 (PMC12251203; doi:10.1371/journal.pone.0326226)
Supplement: S1 Appendix — (DOCX) [file pone.0326226.s001.docx]

**Appendix**

**Table 1** **Weights of the principles and chosen elements used in the integrated index of CPSM.**

| Principles | | Weight*_i_* | Element | Question | Response scale | Weight*_ji_* | | Mean | Std dev | N |
| --- | --- | --- | --- | --- | --- | --- | --- | --- | --- | --- |
|  |  |  |  |  |  | max | min |  |  |  |
| Ⅰ | Dependence | 6 | Knowledge1 | Farmer’s understanding about maize’s dependence on insect pollination | 1-5 | 6.33 | 2.33 | 2.00 | 1.32 | 267 |
|  |  |  | Knowledge2 | Farmer’s understanding about sunflower’s dependence on insect pollination | 1-5 | 7.67 | 4 | 4.67 | 1.17 | 267 |
|  |  |  | Knowledge3 | Farmer’s understanding about melons’ dependence on insect pollination | 1-5 | 9 | 4.33 | 3.10 | 2.35 | 267 |
|  |  |  | Knowledge4 | Farmer’s understanding about other crops’ dependence on insect pollination | 1-5 | 4.33 | 2.67 | 0.14 | 0.33 | 267 |
| Ⅱ | Contribution | 6.67 | Observation1 | Farmers’ observation the frequency of occurrence about the bees’ visits to crops | 1-5 | 8.33 | 5 | 3.93 | 1.05 | 267 |
|  |  |  | Observation2 | Farmers’ observation the frequency of occurrence about the butterflies’ visits to crops | 1-5 | 4.67 | 3 | 2.14 | 1.30 | 267 |
|  |  |  | Observation3 | Farmers’ observation the frequency of occurrence about the flies’ visits to crops | 1-5 | 4.67 | 3 | 3.31 | 1.43 | 267 |
|  |  |  | Observation4 | Farmers’ observation the frequency of occurrence about the ants’ visits to crops | 1-5 | 2.67 | 1 | 2.21 | 1.38 | 267 |
| Ⅲ | Sensitivity | 5.67 | Perception1 | Farmers’ perception of the extent to which yields decline compared to potential yields in the absence of pollinators | 1-5 | 6.33 | 3 | 2.90 | 0.96 | 267 |
|  |  |  | Perception2 | Farmers’ perception of the extent of quality degradation compared to potential quality in the absence of pollinators | 1-5 | 5 | 3 | 2.69 | 0.91 | 267 |
| Ⅳ | Execution | 7 | Practice1 | Install beehives, own beekeeping, or purchase insect pollination services to pollinate crops | 1-5 | 8 | 4 | 1.17 | 0.68 | 267 |
|  |  |  | Practice2 | Beekeepers raise bees around farmland | 1-5 | 8 | 4.67 | 4.55 | 0.89 | 267 |
|  |  |  | Practice3 | Avoid spraying pesticides during flowering period | 1-5 | 8.67 | 2 | 4.47 | 1.03 | 267 |
|  |  |  | Practice4 | Green vegetation retaining farmland boundary | 1-5 | 6.67 | 3.33 | 2.49 | 1.63 | 267 |
|  |  |  | Practice5 | Preserve wildflowers around farmland | 1-5 | 7.33 | 3.33 | 2.32 | 1.53 | 267 |

**Table 2 Descriptive statistics of the elements used to measure the TPB constructs.**

| Index | Elements | Description of question | Response scale | Mean | S. D. | Element loadings | Cronbach’s α value for each construct |
| --- | --- | --- | --- | --- | --- | --- | --- |
| AT | AT1 | Conservation tillage can effectively control weeds | Disagree (1)- Agree (5) | 2.98 | 1.55 | 0.730 | 0.804 |
|  | AT2 | Conservation tillage can effectively control pathogenic microorganisms that can indirectly contribute to pollinator protection |  | 3.31 | 1.63 | 0.888 |  |
|  | AT3 | Conservation tillage can effectively control pests that can indirectly helping to protect pollinators |  | 2.97 | 1.56 | 0.912 |  |
|  | AT4 | Conservation tillage can effectively improve and maintain soil structure and fertility. |  | 4.36 | 1.26 | 0.616 |  |
| SN | SN1 | Other farmers’ actions or suggestions will affect your crop-pollination service management. | Disagree (1)- Agree (5) | 2.31 | 1.73 | 0.795 | 0.751 |
|  | SN2 | The stage of crop growth affects affects your crop-pollination service management. |  | 2.6 | 1.78 | 0.779 |  |
|  | SN3 | Weather conditions will affect your crop-pollination service management. |  | 2.67 | 1.82 | 0.781 |  |
|  | SN4 | The advice of the agricultural technology promotion station will affect your crop-pollination service management. |  | 2.28 | 1.54 | 0.663 |  |
| PBC | PBC1 | Protecting beneficial pollinators. | Disagree (1)- Agree (5) | 4.77 | 0.69 | 0.934 | 0.915 |
|  | PBC2 | Observation of farmland before spraying pesticides. |  | 4.74 | 0.73 | 0.943 |  |
|  | PBC3 | Implement sustainable conservation tillage methods. |  | 4.71 | 0.84 | 0.909 |  |
| EI | EI | Agricultural profit ($) | ≥0 | 5493.50 | 8236.26 |  |  |
| Overall Cronbach’s α | | | 0.787 | | | | |
| KMO | | | 0.773 | | | | |
| Bartlett test of sphericity | | | 0.000 | | | | |

**Table 3 Descriptive statistics of different principles of CPSM.**

| Principle | Min. | Mean | Max. | Std. Dev. |
| --- | --- | --- | --- | --- |
| Contribution | 3.93 | 6.66 | 5.37 | 0.61 |
| Dependence | 2.93 | 6.00 | 4.58 | 0.65 |
| Sensitivity | 3.00 | 5.66 | 4.22 | 0.60 |
| Execution | 3.59 | 6.64 | 5.22 | 0.67 |

**The supplement of the integrated index’s algorithm**

The choice of elements to include in the integrated index is based on elements associated with the four principles of the framework of CPSM as listed in Appendix (Table 1). To ensure the elements are relevant for the Dengkou County, we reviewed literature, conferred with experts, and performed test interviews with members of the survey population. To determine the weights associated with principles and elements, a variant of the Delphi method known as Mini-Delphi (estimate-talk-estimate) was employed (Pan et al., 1996). This approach facilitates a collective assessment of a predetermined set of questions by experts, allowing them to adjust their views through structured discussions. For this study, three experts with extensive knowledge of agricultural pollination in Dengkou County, specifically in pollination management, were consulted by the Dengkou County Agricultural Extension Service. Initially, the experts were individually asked to assign weights to each principle. Subsequently, they were requested to assign weights to the selected elements under each principle based on their perceived importance as indicators of CPSM within the local environment. The experts were instructed to assign a weight of “1” to denote the least important principle and the least important element under each principle. Next, the experts were tasked with ranking all elements under the principles without any upper limit on the weight. This ranking system allowed for relative comparisons, where an element assigned a weight of “2” represented twice the importance of the least important element, and an element with a weight of “3” was considered three times as important as the least important element. Additionally, experts were required to assign two weights to each element, one denoting the importance of maximizing the element, and the other indicating the importance of not enhance the element in the integrated index. The initial evaluation was completed individually by each expert. Subsequently, a meeting was organized to address any potential misunderstandings regarding the project description or wording, discuss the specific aspects of the integrated index, and provide justifications for their weight estimates. Following the meeting, a final round of individual assessments was conducted, and the average weights assigned by the three experts were calculated. These average weights were then utilized in the construction of the integrated index. Weights of the principles and chosen elements used in the integrated index are shown in Appendix (Table 1).

**Algorithm for principles’ scores**

Corresponding principles’ scores are calculated for each principle based on the weighted sum of the selected elements. As shown in Eq. 2,3,4,5.

${\text{P}\text{S}}_{\text{Ⅰ}}\text{=}\sum_{\text{j=}\text{1}}^{\text{4}} \frac{\text{(}\text{Knowledge}_{\text{j}}\text{*}\text{W}_{\text{j}\text{Ⅰ}\text{max}}\text{)}}{\text{Max score of principle}_{\text{Ⅰ}}}\text{+}\frac{\text{(}\text{(}\text{1}\text{-}\text{Knowledge}_{\text{j}}\text{)}\text{*}\text{W}_{\text{j}\text{Ⅰ}\text{min}}\text{)}}{\text{Max score of principle}_{\text{Ⅰ}}}$ (2)

*PS_Ⅰ_* is respondents’ scores of dependence, “*Knowledge_j_*” is scores of *j*th element in dependence. *W_jⅠmax/min_* is max or min weight set for *j*th element in dependence. *W_jⅠmax/min_* can be found in Appendix (Table 1, column 7 and 8).

${\text{P}\text{S}}_{\text{Ⅱ}}\text{=}\sum_{\text{j=}\text{1}}^{\text{4}} \frac{\text{(}\text{Observation}_{\text{j}}\text{*}\text{W}_{\text{j}\text{Ⅱ}\text{max}}\text{)}}{\text{Max score of principle}_{\text{Ⅱ}}}\text{+}\frac{\text{(}\text{(}\text{1}\text{-}\text{Observation}_{\text{j}}\text{)}\text{*}\text{W}_{\text{j}\text{Ⅱ}\text{min}}\text{)}}{\text{Max score of principle}_{\text{Ⅱ}}}$ (3)

*PS_Ⅱ_* is respondents’ scores of contribution, “*Observation_j_*” is scores of *j*th element in contribution. *W_jⅡmax/min_* is max or min weight set for *j*th element in contribution. *W_jⅡmax/min_* could be found in Appendix (Table 1, column 7 and 8).

${\text{P}\text{S}}_{\text{Ⅲ}}\text{=}\sum_{\text{j=}\text{1}}^{\text{2}} \frac{\text{(}\text{Perception}_{\text{j}}\text{*}\text{W}_{\text{j}\text{Ⅲ}\text{max}}\text{)}}{\text{Max score of principle}_{\text{Ⅲ}}}\text{+}\frac{\text{(}\text{(}\text{1}\text{-}\text{Perception}_{\text{j}}\text{)}\text{*}\text{W}_{\text{j}\text{Ⅲ}\text{min}}\text{)}}{\text{Max score of principle}_{\text{Ⅲ}}}$ (4)

PS*_Ⅲ_* is respondents’ scores of sensitivity, “*Perception_j_*” is scores of *j*th element in senitivity. *W_jⅢmax/min_* is max or min weight set for *j*th element in sensitivity. *W_jⅢmax/min_* could be found in Appendix (Table 1, column 7 and 8).

${\text{P}\text{S}}_{\text{Ⅳ}}\text{=}\sum_{\text{j=}\text{1}}^{\text{5}} \frac{\text{(}\text{Practice}_{\text{j}}\text{*}\text{W}_{\text{j}\text{Ⅳ}\text{max}}\text{)}}{\text{Max score of principle}_{\text{Ⅳ}}}\text{+}\frac{\text{(}\text{(}\text{1}\text{-}\text{Practice}_{\text{j}}\text{)}\text{*}\text{W}_{\text{j}\text{Ⅳ}\text{min}}\text{)}}{\text{Max score of principle}_{\text{Ⅳ}}}$ (5)

PS*_Ⅳ_* is respondents’ scores of execution, “*Practice_j_*” is scores of *j*th element in execution. *W_jⅣmax/min_* is max or min weight set for *j*th element in execution. *W_jⅣmax/min_* could be found in Appendix (Table 1, column 7 and 8).

Thirdly, each principle’s score is weighted and summed to obtain the primary integrated index. Calculation formula is shown as Eq. 6.

$\text{Primary\_ICSM}\text{=}\sum_{\text{i}\text{=Ⅰ}}^{\text{Ⅳ}} \text{PS}_{\text{i}}\text{*}\text{W}_{\text{i}}$ (6)

*Primary_ICSM* is respondents’ primary integrated index scores. *W_i_* indicates weight of *PS_i_*, which could be found in Appendix (Table 1, column 3).

**Algorithm for converting the *Primary_ICSM* into the integrated index**

*ICSM* are transformed from *primary_ICSM* to the range of 0-100. Max score of *primary*_*ICSM* is the sum of the weights of principles, which is 25.333. Min score of *primary*_*ICSM* is the sum of the minimal scores of principles, which is 12.999. Algorithm for converting the *primary_ICSM into ICSM* is shown in Eq. 1:

*ICSM*=100**Primary_ICSM* / (25.333-12.999)-12.999 / (25.333-12.999) (1)

Weights of principles and minimal scores of principles could be found in Appendix (Table 1, column 3 and 8)
