# Supplementary material for: Configuration analysis of crop-pollination service management: a novel insight from the theory of planned behavior
Source: PLoS One. 2025 Jul 11;20(7):e0326226. doi: 10.1371/journal.pone.0326226 (PMC12251203; doi:10.1371/journal.pone.0326226)
Supplement: S2 Code — (DOCX) [file pone.0326226.s002.docx]

# R Code

library(readxl)

df <- read_excel("Data.xlsx")

df$lnICSM <- log(df$ICSM)

summary_table <- summary(df[c("Gender", "Education", "Age", "Agricultural_acreage", "EI")])

print(summary_table)

library(broom)

model1 <- lm(lnICSM ~ Gender + Education + Age, data = df)

tidy(model1)

model2 <- lm(lnICSM ~ Gender + Education + Age + AT, data = df)

tidy(model2)

model3 <- lm(lnICSM ~ Gender + Education + Age + SN, data = df)

tidy(model3)

model4 <- lm(lnICSM ~ Gender + Education + Age + PBC, data = df)

tidy(model4)

model5 <- lm(lnICSM ~ Gender + Education + Age + lnEI, data = df)

tidy(model5)

model6 <- lm(lnICSM ~ Gender + Education + Age + AT + SN + PBC + lnEI, data = df)

tidy(model6)

library(NCA)

data <- read.csv("data2.csv")

nca(data,1,5)

nca(data,c(1,4),5)

model1<-nca_analysis(data,1,5,test.rep=1000)

nca_output(model1,summaries=TRUE)

model1$bottlenecks

nca_output(model1, bottlenecks=TRUE)
