# Supplementary material for: Configuration analysis of crop-pollination service management: a novel insight from the theory of planned behavior
Source: PLoS One. 2025 Jul 11;20(7):e0326226. doi: 10.1371/journal.pone.0326226 (PMC12251203; doi:10.1371/journal.pone.0326226)
Supplement: Attachment — Submitted filename: Cooments.pdf [file pone.0326226.s004.pdf]

Comments-RGB

Positives

The study demonstrates a high degree of analytical rigor through its use of multiple advanced statistical and analytical techniques, showcasing the robustness of its methodology. The combination of regression analysis, necessary condition analysis (NCA), fuzzy-set qualitative comparative analysis (fsQCA), and the Mann-Whitney U test provides a multi-faceted approach to examining the factors influencing crop-pollination service management (CPSM). Each method contributes unique insights, enhancing the reliability and depth of the findings.

Regression analysis effectively identifies direct relationships, revealing that education level and agricultural acreage are positively correlated with CPSM. This establishes a clear statistical foundation for understanding key influencing factors. The inclusion of NCA ensures that essential conditions for CPSM are identified, providing a more nuanced perspective on what factors are indispensable. FsQCA adds a configurational perspective, allowing the study to explore combinations of conditions that drive CPSM behaviors, which is particularly valuable for understanding complex, multi-causal phenomena. The Mann-Whitney U test further strengthens the analysis by comparing differences between groups, ensuring the findings are robust across various farmer demographics.

By integrating these diverse analytical approaches, the study not only corroborates its findings across multiple methods but also provides a comprehensive understanding of the dynamics at play. This robust analytical framework enhances the validity of the study's conclusions and supports its practical implications for improving CPSM among farmers. The depth and breadth of the analysis reflect a well-executed research design, making it a significant contribution to the field of sustainable agriculture.

Need for further considerations

However, the study requires major review and some corrections before consideration can be given for publication. These re outlined below:

Suggested issue 1:

The study provides a comprehensive exploration of crop-pollination service management (CPSM) and identifies critical factors influencing farmers' behaviors. It employs a robust methodological framework, grounded in the Theory of Planned Behavior (TPB), and uses a range of analytical techniques such as regression analysis, necessary condition analysis, fuzzy-set qualitative comparative analysis (NCA-fsQCA), and the Mann-Whitney U test. The research reveals that education level and agricultural acreage positively correlate with CPSM and identifies three causal configurations for enhancing CPSM: the AT & PBC path, the AT & Economic Incentive path, and the PBC & Economic Incentive path. Additionally, the study finds contrasting effects of antecedent variables across different principles of CPSM and establishes that the optimal state of CPSM

requires an economic incentive of at least $1900.27. These findings offer practical strategies for improving CPSM and increasing farmer participation in pollinator-supporting behaviors in agricultural practices.

However, the study does not address cultural backgrounds (including local traditions and religion), which are often pivotal in shaping farmers' perceptions, knowledge, and decisions about agricultural practices. Cultural values, traditions, and norms can significantly influence attitudes (AT), perceived behavioral control (PBC), and economic considerations, which are central to the TPB framework used in this research. The omission of cultural factors may limit the depth and applicability of the findings, especially given the rural context of Dengkou County, where traditional beliefs and community practices likely play a role in shaping agricultural behavior.

Explore cultural and regional factors that might influence the applicability of the findings to other settings beyond Dengkou County. To enhance the study, it would be valuable to integrate an analysis of cultural influences on CPSM behaviors. For instance, exploring traditional agricultural practices, beliefs about pollination, and local community norms could provide a richer understanding of farmers' decision-making processes. Incorporating cultural variables into the survey or conducting qualitative interviews to examine these aspects would add depth and nuance to the findings. Such an approach would contextualize the results within the socio-cultural environment of Dengkou County, making the study more robust and actionable. By addressing the role of cultural background, the research could offer a more comprehensive perspective on the factors driving CPSM, ultimately enhancing its theoretical and practical contributions to sustainable agriculture.

Suggestion issue 2:

While the study focuses on socio-economic and behavioral factors, it could benefit from integrating ecological data, such as the diversity and abundance of pollinators in the surveyed farms, to correlate management practices with actual pollination outcome. The study is well-structured and provides valuable insights into CPSM. By incorporating ecological factors, validating tools, and exploring regional differences, the analysis can become even more robust and impactful. These suggestions can enhance the study's ability to inform effective CPSM strategies and policies.

Suggestion issue 3:

A cross-sectional survey provides a snapshot, but a longitudinal approach could better capture changes in farmer behavior over time and the long-term impact of interventions. This may relooked at or justification for exclusion provided

Suggestion issue 4:

The mention of consulting regional pollinator experts and non-academic stakeholders in the discussion section, rather than the methodology, is a notable issue. It creates a gap in the transparency of the research process. If these consultations played a role in constructing the framework, they should have been explicitly described in the methodology to clarify how these insights influenced the study's design.

To improve, the consultation process should be added to the methodology section, detailing how experts and stakeholders contributed to framework development. Additionally, the influence of these consultations on the research outcomes should be explicitly stated, ensuring the study reflects a comprehensive and well-informed approach to the ecological context.

I strongly recommend relocating the following section from the supplementary materials to the methods section, as it is essential to the study and directly supports the extensive deliberations presented in the discussion and also presented in data analysis:

"To ensure the elements are relevant for Dengkou County, we reviewed literature, conferred with experts, and performed test interviews with members of the survey population. To determine the weights associated with principles and elements, a variant of the Delphi method known as Mini-Delphi (estimate-talk-estimate) was employed (Pan et al., 1996). This approach facilitates a collective assessment of a predetermined set of questions by experts, allowing them to adjust their views through structured discussions. For this study, three experts with extensive knowledge of agricultural pollination in Dengkou County, specifically in pollination management, were consulted by the Dengkou County Agricultural Extension Service."

Including this section in the methods would provide much-needed clarity on how the principles and weights were derived, which is central to the study's framework. Furthermore, its prominent role in the discussion underscores its importance, making its inclusion in the methods section critical for readers to fully understand and contextualize the findings. This move would enhance the study's transparency, methodological rigor, and alignment between the methods and discussion sections.

Suggested issue 5: Critique of placement in main text vs. supplementary material

1. Relevance to core methodology
   Equations like these are critical to understanding the methodology used to calculate scores and indices central to the study. They detail how the analysis is conducted and provide transparency, ensuring replicability. However, they are dense and mathematical, which might distract readers from the broader methodological narrative if presented in the main text.

2. Purpose of the equations

    - If the equations are essential for readers to follow and evaluate the results and methodology, they should remain in the main text.
    - If they are primarily supporting details for replicability or validation purposes, they could be moved to supplementary material.
3. Accessibility for the audience
    - For a broad audience, including practitioners and policymakers, presenting such detailed equations in the main text might reduce readability.
    - For a more specialized audience, such as academic researchers familiar with these methods, keeping them in the main text might be justified.

4. Current context
    - The equations are highly detailed and seem to be supported by a reference to the appendix, where additional data and variables (e.g., weights, algorithms) are described. This redundancy suggests they could be summarized in the main text with a clear reference to supplementary material for those seeking further detail.

Recommendation: balanced approach

- Main Text: Provide a concise summary of the methodology, outlining the key steps in plain language. Include one representative equation (e.g., Eq. 1) to illustrate the process, but avoid including all equations.
- Supplementary Material: Move the detailed equations (Eqs. 2-6) and algorithmic descriptions to the supplementary material, as they are technical details that support the core methodology but are not critical for the broader understanding of the study.

Suggested rewrite for main text

In the main text, you could say:
"To calculate the scores for each principle, we summed the maximum points of the elements within each principle and computed the corresponding scores based on weighted sums of the selected elements (Eq. 1). This process involved calculating respondents' scores for dependence, contribution, sensitivity, and execution using weighted principles and elements (detailed in Supplementary Material, Eqs. 2-6). These scores were then aggregated to obtain the Primary ICSM and subsequently converted to ICSM scores ranging from 0 to 100 (see Supplementary Material for the complete algorithm)."

Suggested issue 6:

The authors introduced the **Theory of Planned Behavior (TPB)** and its extension using economic incentives (EI) as a framework for analyzing crop-pollination service management (CPSM). However, their explanation lacks a thorough introduction and context for readers unfamiliar with TPB. The explanation of the Theory of Planned Behavior (TPB) in the manuscript is inadequate, lacking a thorough introduction to its core concepts. Key components like attitude (AT), subjective norm (SN), and perceived behavioral control (PBC) are briefly mentioned without sufficient

context or real-world examples, making it inaccessible to readers unfamiliar with the theory. Additionally, the manuscript introduces complexity theory in the section title but fails to elaborate on it, causing confusion. The inclusion of economic incentives (EI) to extend TPB is not adequately justified, as assumptions about farmers' motivations lack supporting evidence. The section's structure is disorganized, moving abruptly between concepts without a clear link. To improve, the authors should explain TPB in detail, justify its relevance to crop-pollination management, and integrate economic incentives with supporting data.

Suggested issue 7:

The manuscript is littered with many sentences which require rewording to bring clarity. I have listed some below and follow up is required on others:

Line 169: The sentence "Contribution refers to farmers' observation the frequency of occurrence about pollinator's visits to crops" is not well-worded and needs clarification for proper grammatical structure and meaning. Here's a suggested revision: "Contribution refers to farmers' observations of the frequency of pollinator visits to crops"

Line 54: The sentence "Moreover, pollinator-dependent crops show higher agricultural expansion rates than other crops" is not well-worded and needs clarification for proper grammatical structure and meaning. Here's a suggested revision: "Suggestion: Additionally, crops that depend on pollinators exhibit higher rates of agricultural expansion compared to non-pollinator-dependent crops"

Line 86: The phrase "it to a large extent has higher potential" is awkward and not grammatically sound. It needs rephrasing for clarity and flow

Line 183: The sentence "The more diversified are farmers' adoption pollinator-supporting practices, the more options they must avoid adverse effects induced by inadequate contribution, optimizing discrepancies between farmers' cognition of pollinator dependence and researchers" needs rewording. Suggested re-wording: "The more diversified farmers' adoption of pollinator-supporting practices, the greater their options for avoiding adverse effects caused by insufficient pollinator contributions. This approach also helps to optimize the discrepancies between farmers' perceptions of pollinator dependence and those of researchers"

Line 268: The sentence "Among these crops, sunflower and melon are more rely on pollination mediated by insects" needs rewording. Suggested rewording "Among these crops, sunflower and melon rely more on pollination mediated by insects."

Line 283: The sentence "**The start and end dates of the recruitment period for this study are 01/07/2021 and 31/08/2021, respectively."** is clear but could be improved for readability and formality. Suggested revision: "The recruitment period for this study began on July 1, 2021, and ended on August 31, 2021."

Line 76-81 To avoid repetitive use of 'on the other hand in two sentences following each other immediately, consider this ["While differences in perceptions between farmers and researchers or policymakers highlight an understanding and communication gap (Batie et al., 2009), these disparities also reflect variations in knowledge and background among local farmers. This is because the benefits of pollinator-supporting practices on yield can vary depending on management factors, and farmer behavior—an essential component of agricultural practices—is directly influenced by their local knowledge and experience (Batie et al., 2019)]

Line 308: The sentence "However, the measurement farmers' CPSM, which should attempt to consider specific regions and various crop types, is very difficult" is not clear. The sentence structure could be simplified for better readability. A more polished revision would be: "However, measuring farmers' CPSM, which should account for specific regions and various crop types, is challenging."

Line 663: The sentence "The enforcement agencies of pollination compensation will be refined to ensure the strict enforcement of pollination protection laws, and the central legislation 664 will be coordinated with the implementation of local departments" needs refinement. **Suggested revision** "The enforcement agencies responsible for pollination protection should be strengthened to ensure strict adherence to pollination protection laws, and coordination between national legislation and local departments should be improved to support effective implementation."

Line 583: [Farmers more affected by SN are less sensitive to the value of CPSM 582 in regulating crop yield and quality, therefore, SN does not support CPSM for farmers in Dengkou County (Hipólito et al., 2018).] **Suggestion revision** "Farmers who are more affected by SN are less sensitive to the value of CPSM in regulating crop yield and quality. Therefore, SN does not support CPSM for farmers in Dengkou County (Hipólito et al., 2018)."

Suggested issue 8: Line 269: In the methodology,

The sentence "unwritten regulations" may cause confusion, as it suggests rules that are not formally documented, which can be ambiguous in academic writing. To clarify, it's better to use "informal regulations" if referring to practices that are followed but not codified. Additionally, the sentence uses "enough" twice, which makes it sound repetitive. To improve readability and flow, varying the language would help. The contraction "don't" should also be avoided in formal writing, replaced with "do not" for a more professional tone. Lastly, the sentence structure could be refined to ensure greater clarity and formality. A revised version would be: "However, policies and informal regulations in Dengkou County do not give sufficient attention to crop-pollination services, and farmers lack adequate awareness of CPSM.

Suggested issue 9:

Line 290: The passage provides important ethical information but lacks clarity and coherence. The phrase "this study did not involve human or animal experimentation" is unnecessary since it contradicts earlier mentions of human participants. It would also benefit from clearer explanation about how informed consent was obtained and how respondents' data was protected. **Suggested revision:** "The Ethics Committee of the School of Economics and Management, Inner Mongolia Normal University, Hohhot, China, approved this study. All procedures involving human participants adhered to ethical standards, including the Declaration of Helsinki. Informed consent was obtained from all participants, and data were collected securely via online software to protect personal information."

Suggested issue 10: The phrase "267 questionnaires are returned" should be rephrased to improve readability, and the connection between the questionnaires and other data sources should be more clearly established. **Suggested revision: "**A total of 267 completed questionnaires were returned, all of which were deemed valid. The questionnaire covered elements related to CPSM and TPB, with specific questions listed in the Appendix (Table 1: Weights of the principles and chosen elements used in ICSM; Table 2: Descriptive statistics of the elements used to measure TPB constructs).

Suggested issue 11:

Placement of dollar sign not consistent. The dollar sign in the sentence is not correctly placed. It should come before the number for consistency with standard formatting. Example below [Pollination compensation standard in Dengkou County should be 152.27$, which is the lowest EI ($1900.27) of the optimal CPSM minus the difference between farmers' income and expenditure.]

Suggested issue 12:

Check to see if the following were meant to be heading or sentences

- Motivate CPSM by improving farmers' AT about pollinator
- Innovate and popularize technology about CPSM
