# Supplementary material for: Configuration analysis of crop-pollination service management: a novel insight from the theory of planned behavior
Source: PLoS One. 2025 Jul 11;20(7):e0326226. doi: 10.1371/journal.pone.0326226 (PMC12251203; doi:10.1371/journal.pone.0326226)
Supplement: Attachment — Submitted filename: Review_Report_Richard_Boakye.pdf [file pone.0326226.s006.pdf]

Review Report for Manuscript: PONE-D-24-49557_R1
(By Dr Richard Gyamfi Boakye)

This report evaluates the revisions made by the authors in response to the previous recommendations for the manuscript titled '**Configuration analysis of crop-pollination service management: A novel insight from the theory of planned behavior'.** The recommendations made in the earlier manuscript are assessed to determine if they were properly addressed in the revised manuscript.

1. Cultural Factors

- *Previous Recommendation:* The manuscript should explore cultural influences on farmers' decision-making processes, including beliefs, traditions, and community norms, particularly in rural contexts like Dengkou County.
- *Revised Response:* The authors acknowledged the importance of cultural factors and stated that while subjective norms in the Theory of Planned Behavior (TPB) reflect social and cultural influences, the study did not delve deeply into cultural background due to funding limitations. The authors have committed to incorporating these cultural factors in future studies.
- *Assessment:* The suggestion has not been fully implemented, but the authors have recognized its significance and proposed addressing it in future research. This acknowledgement is deemed satisfactory.

2. Ecological Data

- *Previous Recommendation:* The authors were advised to integrate ecological data, such as pollinator diversity and abundance, to correlate management practices with actual pollination outcomes.
- *Revised Response:* The authors explained that the study focused on socio-economic and behavioral factors, aligning with the scope of their research objectives. They acknowledged that ecological data would be a valuable addition in future research.
- *Assessment:* The recommendation was acknowledged, but the suggestion was not fully implemented due to study constraints. I acknowledge that and express satisfaction.

3. Longitudinal Approach

- Previous Recommendation: A longitudinal study could offer deeper insights into changes in farmers' behavior over time and the long-term impacts of interventions.
- Revised Response: The authors maintained that their study used a cross-sectional design, which was appropriate for their research objectives. They expressed intent to explore a longitudinal approach in future research.
- Assessment: The authors justified the exclusion of the longitudinal approach and did not implement it in the revised manuscript. Their justification is satisfactory.

4. Consultation Process with Experts

- *Previous Recommendation:* The consultation with regional pollinator experts and non-academic stakeholders should be explicitly described in the methodology section for clarity and transparency.
- *Revised Response:* The authors agreed with this suggestion and moved the description of expert consultations from the supplementary materials to the methodology section. They also clarified how these consultations contributed to the development of the study's framework.
- *Assessment:* This recommendation has been fully addressed, and the consultation process is now clearly stated in the methodology section.

5. Equations and Methodology

- *Previous Recommendation:* The detailed equations used in the study should be simplified in the main text, with supporting materials placed in the supplementary section.
- *Revised Response:* The authors followed this recommendation by summarizing the methodology in plain language in the main text and placing the detailed equations in the supplementary material.
- *Assessment:* The authors have implemented this recommendation effectively.

6. Theory of Planned Behavior (TPB)

- *Previous Recommendation:* A more thorough introduction of the TPB and its extension with economic incentives should be provided for readers unfamiliar with the theory.
- *Revised Response*: The authors expanded their explanation of the TPB, including more context to make the theory accessible to a broader audience. They also clarified the extension of TPB using economic incentives.
- *Assessment:* The authors have addressed this recommendation in the revised manuscript.

7. Suggested revision of sentences

Generally, all sentenced needing revision have been revised accordingly

Overall Recommendation for Publication

Based on the revisions made by the authors, most of the suggestions were considered, especially the inclusion of expert consultations in methodology and the restructuring of the equations. However, some points, such as incorporating cultural influences and ecological data, were either beyond the scope of the current study or planned for future research. Given these revisions, I recommend acceptance for publication. The paper demonstrates significant improvements, particularly in methodological transparency, but could benefit from the integration of cultural and ecological factors in future work.
