# Supplementary material for: Configuration analysis of crop-pollination service management: a novel insight from the theory of planned behavior
Source: PLoS One. 2025 Jul 11;20(7):e0326226. doi: 10.1371/journal.pone.0326226 (PMC12251203; doi:10.1371/journal.pone.0326226)
Supplement: Attachment — Submitted filename: Response to Reviewers.docx [file pone.0326226.s005.docx]

**List of Responses**

**Dear Editors and Reviewers:**

Thank you for your letter and for the reviewer’s comments concerning our manuscript entitled “Configuration analysis of crop-pollination service management: a novel insight from the theory of planned behavior” (Submission ID: PONE-D-24-49557). These comments are all valuable and very helpful for revising and improving our manuscript, as well as the important guiding significance to our researches. We have studied comments carefully and have made correction which we hope meet with approval. The main corrections in the manuscript and the responds to the reviewer’s comments are as flowing:

**Revision note:**

In the detailed responses to the reviewers listed below, the black lines are the reviewers’ comments and the blue lines are our responses.

# Response to reviewer 1:

**Issue 1.:** However, the study only analysed the impact of farmers on the functioning of crop pollination systems without understanding the underlying logic of pollination systems, and did not take into account the natural ecological environment, anthropological farming practices, changes in crop species, background and changes in pollinator diversity, and ecological factors affecting pollination as directly relevant to the pollination system, and lacked analysis of the basic knowledge of the underlying biological systems to support it. There is also no analysis of the impact on pollinators of a major human action - changes in large-scale, man-made agriculture.

Thank you for your detailed feedback. We appreciate your insights regarding the scope of our study. However, we would like to clarify the focus and objectives of our research.

Our study primarily aims to explore farmers' perceptions, knowledge, and decision-making behaviors regarding crop-pollination service management (CPSM), rather than conducting an ecological or biological analysis of pollination systems. Given that farmers are the direct decision-makers in agricultural management, understanding their behavior and the factors influencing their CPSM practices is crucial for developing effective policies and interventions.

While we acknowledge the importance of ecological factors such as pollinator diversity, environmental changes, and large-scale agricultural modifications, these aspects were beyond the scope of our research. Instead, we focused on behavioral and socioeconomic determinants, as grounded in the extended Theory of Planned Behavior (TPB), to identify actionable strategies for improving CPSM at the farmer level.

Furthermore, our study provides empirical evidence through regression analysis, necessary condition analysis (NCA), fuzzy-set qualitative comparative analysis (fsQCA), and the idependent smples t-test, demonstrating how key socioeconomic and psychological factors shape farmers’ pollination management behaviors. These insights are directly relevant to agricultural policy and practice, complementing existing ecological research by offering practical strategies to enhance farmers’ participation in pollinator-supporting behaviors.

We appreciate your suggestion to incorporate broader ecological perspectives, and we acknowledge their relevance to the field of pollination research. However, integrating such analyses would require a different database, research design and methodology, which goes beyond the behavioral and management-focused objectives of this study.

We hope this clarification addresses your concerns, and we sincerely appreciate your thoughtful review.

**Issue 2.:** introduction should be supplemented with the type and species of pollinators, the part did not pay attention to the flowering period of crops, other plants and pollinators change information, this part of the content is closely related to pollination, it is the main factors that lead to anthropogenic influence on pollination system, these contents will lead to changes in the concept of pollination system of farmers of the main content. This part was missing in the study, which may directly lead to the wrong direction of the study.

Thank you for your insightful comments. We recognize the significance of pollinator species, flowering periods, and ecological changes in pollination systems. However, our study primarily focuses on farmers’ perceptions and behaviors regarding crop-pollination service management (CPSM) through the lens of the extended Theory of Planned Behavior (TPB). Our objective is to explore how factors such as farmers’ knowledge, attitudes, economic incentives, and farm size influence their CPSM practices. While ecological aspects do influence farmers’ experiences, our research centers on how they manage pollination services, rather than the biological mechanisms behind it.

Still, we appreciate your suggestion to supplement the introduction with more detailed information on the types and species of pollinators. We revised the introduction section to include a broader discussion on the diversity of pollinators, such as wild insects, artificial pollinators, and their respective roles in the pollination process.

**Introduction:**

Crop-pollination is a vital ecosystem service required by many crops, particularly those that rely on insect pollination, such as sunflowers and melons. Historically, natural crop-pollination by wild pollinators such as bees, butterflies and flies has played a central role in maintaining agricultural productivity and biodiversity (Aizen et al., 2009). This method of pollination, rooted in local ecosystems, is sustainable but vulnerable to threats such as habitat loss, pesticide use and climate change, which can reduce pollinator diversity and stability (Chagnon et al., 2015). With the advent of industrial agriculture, crop-pollination has become increasingly dependent on human intervention, particularly through the use of managed pollinators such as honey bees (Wurz et al., 2021). While this shift has enabled higher yields and greater efficiency, it has also led to reduced resilience as monoculture practices and pesticide use undermine pollinator diversity. In addition, over-reliance on a few pollinator species in industrial agriculture limits the overall stability of pollination systems (Kovács-Hostyánszki et al., 2017). In response, artificial insect pollination techniques, such as the use of commercially reared pollinators or mechanical pollination, have been introduced in areas where natural pollinators are insufficient (Hayo and Adriaan, 2006). However, these methods are often more expensive and may not provide the same ecological benefits as wild pollination. Balancing these systems while protecting and enhancing natural pollination services will be key to meeting growing environmental challenges and increasing agricultural demands. (Line 63-79)

References:

Aizen, M.A., Garibaldi, L.A., Cunningham, S.A., Klein, A.M., 2009. How much does agriculture depend on pollinators? Lessons from long-term trends in crop production. Annals of Botany 103, 1579-1588.

Chagnon, M., Kreutzweiser, D., Mitchell, E.A.D., Morrissey, C.A., Noome, D.A., Van der Sluijs, J.P., 2015. Risks of large-scale use of systemic insecticides to ecosystem functioning and services. Environmental Science and Pollution Research 22, 119-134.

Wurz, A., Grass, I., Tscharntke, T., 2021. Hand pollination of global crops – A systematic review. Basic and Applied Ecology 56, 299-321.

Kovács-Hostyánszki, A., Espíndola, A., Vanbergen, A.J., Settele, J., Kremen, C., Dicks, L.V., 2017. Ecological intensification to mitigate impacts of conventional intensive land use on pollinators and pollination. Ecology Letters 20, 673-689.

Hayo, H.W.V., Adriaan van, D., 2006. A century of advances in bumblebee domestication and the economic and environmental aspects of its commercialization for pollination. Apidologie 37, 421-451.

**Issue 3.:** Research should also focus on the diversity and variability of flowering times and pollinators of etiolated crops and other plants, and the reasons for this variability.

Thank you for your valuable feedback. We acknowledge the significance of flowering time diversity, pollinator variability, and their underlying causes in understanding pollination dynamics. However, our study primarily focuses on farmers’ perceptions and behaviors in crop-pollination service management (CPSM), rather than the ecological variability of pollinators and flowering periods.

Our research aims to explore socioeconomic and behavioral factors influencing CPSM among farmers, using an approach grounded in the extended theory of planned behavior. While the ecological aspects of pollination are important, they fall beyond the intended scope of this study. Instead, our findings contribute to practical strategies for enhancing farmers’ engagement in pollinator-supporting behaviors.

That said, we appreciate your suggestion and revised the framework of CPSM to better address the variability in flowering times and pollinators. Specifically, we will highlight the role of different pollinator species—such as ants with wings, bees, butterflies, and flies—and their interactions with crops during different flowering periods. Additionally, we will discuss factors contributing to variability, such as crop types. This revision will enhance the understanding of how these variables affect pollinator activity and CPSM, providing a more comprehensive view of the dynamics in pollination systems.

**Section 2. 1 A general framework crop-pollination service management**

Here, the framework of CPSM is provided, which allows farmers to have greater flexibility in pollinator-supporting behaviors to adapt specific landscape conditions, crop varieties, and management strategies. The framework of CPSM, which outlines how farmers trust in the beneficial effects of pollinator-supporting behaviors based on pollination knowledge, linked with their involvement in pollinator-supporting practices, is presented to promote pollinator conservation and enhance crop-pollination service. The four underpinning principles of the framework of CPSM are respectively dependence, contribution, sensitivity, and execution (Fig. 1). To better understand the framework of CPSM, the concepts of principles and the hypothesized links among them are explored below. Dependence represents farmers’ cognition of the pollination dependency of their crops (Fig. 1 Principle I). The pollinator dependence of different crops differs greatly in extent which range from little or no dependence (e.g., wind-pollinated or self-pollinated cereals) to partially dependence (e.g., melon, tomato, and sunflower) and are intrinsically linked to plant breeding systems (Delgado-Carrillo et al., 2015., Shivanna, 2015). Contribution refers to farmers’ observations of the frequency of pollinator visits to crops (Fig. 1 Principle Ⅱ). The frequency of occurrence for pollinator’s visits to crops is often context-dependent on specific landscape conditions and management strategies. Farmers’ observations on the contribution of pollinator-supporting behaviors are often essential to enhance farmers’ knowledge and insights about dependence (Fig. 1 arrow A). Sensitivity stands for farmers’ perceptions of the extent of yield or quality reduction in absence of pollinators compared to potential yield or quality (Fig. 1 Principle Ⅲ). In general, a higher dependency on crop-pollination service could improve farmers’ understanding of sensitivity in CPSM (Fig. 1 arrow B). Indeed, sensitivity might also be relevant to potential effects of contribution changes on farmers’ CPSM, and substituted technologies and other means (such as hand pollination) that farmers implemented in their fields (Fig. 1 arrow C). Execution indicates farmers’ adoption of agricultural practices that promote pollinator conservation and enhance crop-pollination service (pollinator-supporting practices) (Fig. 1 Principle Ⅳ). Farmers’ cognition of sensitivity is important for execution because it may affect individuals’ perception of the effectiveness of adaptive actions, allowing farmers to take diverse pollinator-supporting practices in different CPSM scenarios (Fig. 1 arrow D). The more diversified farmers’ adoption of pollinator-supporting practices, the greater their options for avoiding adverse effects caused by insufficient pollinator contributions. This approach also helps to optimize the discrepancies between farmers’ perceptions of pollinator dependence and those of researchers (Fig. 1 arrow E). At the same time, farmers’ willingness to adopt pollinator-supporting practices also is often limited by their knowledge of dependence (Fig. 1 arrow F). Here, the framework of CPSM highlights a positive, and often neglected, feedback loop of the relationship between farmers’ knowledge and experience and their pollinator-supporting behaviors (Fig. 1 the direction of blue and green arrows). The four principles (dependence, contribution, sensitivity, and execution) within the positive feedback loop of the framework of CPSM may reinforce each other, offering insights into how to implement on-farm CPSM that are more realistic and more suited to local agriculture landscapes. Because the framework of CPSM is built to explore how farmers manage crop-pollination service, it is necessary to assess on-farm CPSM among farmers by using the integrated index based on the framework of CPSM. (Line 172-212)

Fig. 1 A summarized general framework of CPSM. The boxes: four underpinning principles of the framework of CPSM; A-F arrows: hypothesized links among four principles (the direction of blue arrows represents increasing farmers’ trust in pollinator-supporting behaviors based on their pollination knowledge and experience; the direction of green arrows represents farmers’ involvement in pollinator-supporting practices to enrich their related knowledge and experience). For further explanation of principles and links see text.

**Issue 4.****:** Study should complement the development history, current status and importance, differences, advantages and disadvantages, development trends and problems of traditional natural crop pollination, crop pollination in industrial agriculture and crop pollination by insects and other professional pollinators. Based on the results of this study, what measures should be taken to protect the way pollinators pollinate crops?

Thank you for your constructive suggestions. We revised the introduction to include a discussion on the development history, current status, and importance of traditional natural crop pollination, as well as crop pollination in industrial agriculture. We also address the differences, advantages, and disadvantages of these approaches, alongside the role of professional pollinators, such as insects. This revision enriches the context of our study and better aligns it with your valuable input.

Introduction:

Crop-pollination is a vital ecosystem service required by many crops, particularly those that rely on insect pollination, such as sunflowers and melons. Historically, natural crop-pollination by wild pollinators such as bees, butterflies and flies has played a central role in maintaining agricultural productivity and biodiversity (Aizen et al., 2009). This method of pollination, rooted in local ecosystems, is sustainable but vulnerable to threats such as habitat loss, pesticide use and climate change, which can reduce pollinator diversity and stability (Chagnon et al., 2015). With the advent of industrial agriculture, crop-pollination has become increasingly dependent on human intervention, particularly through the use of managed pollinators such as honey bees (Wurz et al., 2021). While this shift has enabled higher yields and greater efficiency, it has also led to reduced resilience as monoculture practices and pesticide use undermine pollinator diversity. In addition, over-reliance on a few pollinator species in industrial agriculture limits the overall stability of pollination systems (Kovács-Hostyánszki et al., 2017). In response, artificial insect pollination techniques, such as the use of commercially reared pollinators or mechanical pollination, have been introduced in areas where natural pollinators are insufficient (Hayo and Adriaan, 2006). However, these methods are often more expensive and may not provide the same ecological benefits as wild pollination. Balancing these systems while protecting and enhancing natural pollination services will be key to meeting growing environmental challenges and increasing agricultural demands. (Line 63-79)

References:

Aizen, M.A., Garibaldi, L.A., Cunningham, S.A., Klein, A.M., 2009. How much does agriculture depend on pollinators? Lessons from long-term trends in crop production. Annals of Botany 103, 1579-1588.

Chagnon, M., Kreutzweiser, D., Mitchell, E.A.D., Morrissey, C.A., Noome, D.A., Van der Sluijs, J.P., 2015. Risks of large-scale use of systemic insecticides to ecosystem functioning and services. Environmental Science and Pollution Research 22, 119-134.

Wurz, A., Grass, I., Tscharntke, T., 2021. Hand pollination of global crops – A systematic review. Basic and Applied Ecology 56, 299-321.

Kovács-Hostyánszki, A., Espíndola, A., Vanbergen, A.J., Settele, J., Kremen, C., Dicks, L.V., 2017. Ecological intensification to mitigate impacts of conventional intensive land use on pollinators and pollination. Ecology Letters 20, 673-689.

Hayo, H.W.V., Adriaan van, D., 2006. A century of advances in bumblebee domestication and the economic and environmental aspects of its commercialization for pollination. Apidologie 37, 421-451.

**Issue 5.:** There are flaws in the content of the study that leads to problems with the design of the study. ‘Contribution is the frequency of occurrence of farmers observing pollinator visits to the crop’, this part of the study is inadequate in terms of the contribution of the pollinators and inadequate for the education of the farmers. This is a flaw in the overall study, and the success and significance of the study is directly determined by the correct and sound choice of research components.

Thank you for your insightful feedback. We appreciate your attention to the design of our study, particularly the definition of “contribution” as the frequency with which farmers observe pollinator visits to their crops. We understand your concern regarding the adequacy of this measure in reflecting the full contribution of pollinators to agricultural productivity, as well as its implications for farmer education.

We would like to clarify that the choice of using farmers’ observations of pollinator visits is intentional and grounded in both the Theory of Planned Behavior (TPB) and agriculture’s practical realities. Farmers’ perceptions and observations are often a primary source of knowledge about the ecological services provided by pollinators. This is especially important in smallholder farming contexts, where formal education on pollinator behavior may be limited, and much of the understanding comes from direct experience and observations in the field. In our study, the frequency of observing pollinator visits serves as a proxy for farmers’ awareness and their perceived importance of pollinators, which has been shown to influence their engagement in pollinator-supporting behaviors.

We fully acknowledge that observations alone may not capture the entire scope of pollinator contribution, particularly the unseen, indirect impacts of pollinators on crop yield and quality. However, our study does not rely solely on this measure but integrates a broader framework through the use of the TPB, which also includes attitudes (AT), perceived behavioral control (PBC), and economic incentives. By evaluating farmers’ perceptions of pollinator support and behaviors, we aim to understand the broader factors that influence their adoption of CPSM strategies. This also aligns with the notion that farmers’ knowledge, education, and role in pollination management are essential to improving pollinator conservation practices on farms.

Regarding the concern about farmer education, we argue that the current study does not neglect this aspect. Rather, it emphasizes how farmers’ observations are linked to their knowledge and the broader CPSM behaviors, particularly through economic incentives and educational pathways. Our study found that education level and agricultural acreage were positively correlated with CPSM, indicating that increasing awareness and education about pollinators can enhance management practices. However, we also recognize that farmer education on the full biological and ecological contribution of pollinators could be further integrated into the study. This could indeed add depth, and we expanded on educational interventions or strategies for improving farmers’ understanding of pollinator services in the revised manuscript.

In conclusion, we believe that our approach of assessing farmers’ perceptions and linking them to CPSM behaviors provides valuable insights into the complex relationship between farmers and pollinator support. While the frequency of observing pollinator visits may not capture every dimension of pollination service, it serves as a meaningful indicator of farmers’ awareness and engagement in supporting pollinators.

We hope this clarification addresses your concerns and improves the overall contribution of the study.

**Issue 6.:** Methodological component: In Dengkou County, maize (53.08%), sunflower (31.8%), melon (1.5%) and a few other crops (3.62%) are grown. Among these crops, sunflower and melon are more dependent on insect pollination. The lack of information on changes in the flowering period and pollinators of these crops and other plants closely related to pollination are the main factors leading to anthropogenic influences on the pollination system, and even more to changes in farmers' perceptions of the pollination system.

Thank you for your valuable advice. We added the information on changes in the flowering period and pollinators of these crops and other plants closely related to pollination.

In Dengkou County, corn (account for 53.08%), sunflower (account for 31.8%), melons (account for 11,5%) and a few other crops (account for 3.62%) are grown. Among these crops, sunflower and melon rely more on pollination mediated by insects. Corn and sunflower bloom in July and August, while melons bloom in late spring and summer. Pollinators are mainly bees, butterflies and flies, of which there are two types, wild bees and domestic bees. (Line 309-313)

# Response to reviewer 2:

**Issue 1. 1.:** Introduction can be more concise, by focusing on the introduction of CPSM, the current limitations in measuring CPSM, following by introducing the concept of TPB and the research gaps. The text should be shortened, especially when section 2 provided more detailed background and hypotheses, and both sections reached to more than 30% of the manuscript. Some of the text can be moved to the Appendix.

Thank you for your suggestion. We understand the need for a more concise introduction, and we agree that focusing on CPSM, its limitations, and introducing TPB and research gaps would improve clarity. However, we also received feedback from other reviewers that prompted us to expand certain sections. As a result, it has been challenging to further condense the manuscript or move content to the Appendix without affecting the overall coherence. That said, we made efforts to streamline the introduction while maintaining the necessary details. We hope the revised version strikes a better balance between conciseness and comprehensiveness.

**Issue 1. 2.:** There are many abbreviations in the text, the authors are recommended to reconsider the usage of abbreviations instead of introducing too many new abbreviations, e.g., based on the first abbreviation CPSM, the ICSM can be written as “integrated index of CPSM”, and FCPM can be written as “framework of CPSM”, etc.

Thank you for your comments and suggestions. We have made the following improvements: (1) Reduce the use of acronyms. We have minimized the use of acronyms in the text by presenting some important terms in their full form to improve readability and comprehension (e. g. ICSM is written as “integrated index of CPSM”, write FCPM is be written as “framework of CPSM”). (2) Provide full name descriptions. For acronyms that must be used, we have provided full names when they first appear and added short descriptions where appropriate to ensure that readers can easily understand their meaning. We believe that these improvements will make the articles clearer and more understandable.

Table 1 List of abbreviations. (Line: 168)

| NCA | Necessary condition analysis |
| --- | --- |
| fsQCA | Qualitative Comparative Analysis of Fuzzy Sets |
| TPB | Theory of planned behavior |
| AT | Attitudes |
| SN | Subjective norm |
| PBC | Perceived behavioral control |
| EI | Economic Incentive |

**Issue 1. 3.:** L50: “about 75% of global crops” varied in their dependence on pollinators, please check the review: “Importance of pollinators in changing landscapes for world crops” from Klein et al. (2007) and add the citation.

Thank you for pointing this out. I revised the sentence to include the citation to Klein et al. (2007) as suggested, as it is a key reference for the statistic about the dependence of global crops on pollinators. Here is the revised sentence:

In context of high human demands for different crops due to population growth and changing dietary preferences, about 35% of global crops that provide nutrients rely on insect-mediated pollination (Garibaldi et al., 2013; Klein et al., 2007; Jordan et al., 2021). (Line: 48-51)

Klein, A.-M., Vaissiere, B.E., Cane, J.H., Steffan-Dewenter, I., Cunningham, S.A., Kremen, C., Tscharntke, T., 2007. Importance of pollinators in changing landscapes for world crops. Proceedings. Biological sciences 274, 303-313.

**Issue 1. 4.:** L80: farmer behavior farmers’ behavior

Thank you for your advice. We modified farmer behavior to farmers’ behavior.

While differences in perceptions between farmers and researchers or policymakers highlight an understanding and communication gap (Batie et al., 2009), these disparities also reflect variations in knowledge and background among local farmers. This is because the benefits of pollinator-supporting practices on yield can vary depending on management factors, and farmers’ behavior—an essential component of agricultural practices—is directly influenced by their local knowledge and experience (Batie et al., 2019). (Line: 93-98)

**Issue 1. 5.:** L86-94: these sentences can be shortened.

Thank you for your suggestion to shorten the sentences in lines 86–94. We shortened these sentences.

Additionally, what proposes the framework of CPSM is also a desire to promote a novel method for quantifying the farmers’ on-farm pollination management to implement multiple pollinator-supporting practices and enhance cooperation between them on landscape scale. Since the framework of CPSM is a comprehensive framework with a view of multi-dimensional realities, measuring farmers’ CPSM based on the framework of CPSM will be important and challenging (Roquer-Beni et al., 2021). (Line: 103-108)

**Issue 1. 6.:** L82, 86: FCPM or FCPSM? As in L64, CPSM was the abbreviation of crop-pollination service management. It can be written as “framework of CPSM”.

Thank you for your advice. We modified the expressions.

Therefore, a general framework of CPSM with greater flexibility for farmers in pollinator-supporting behaviors should be developed, accounting for the trust (related to enhancing confidence in pollinator-supporting behaviors of farmers through understanding of pollination knowledge) and engagements (related to enhancing the trust to actively involve farmers in pollinator-supporting practices). (Line: 98-102)

Since the framework of CPSM is intrinsically linked to knowledge and background of local farmers, it to a large extent has higher potential for farmers to implement multiple pollinator-supporting practices and enhance cooperation between them on landscape scale. (Line: 105-108)

**Issue 1. 7.:** L101: “Index of crop-pollination service management” could be written as “index of CPSM” instead of ICSM and why not use “ICPSM”? Please reconsider the abbreviations used in the text. It can be confusing for readers with many abbreviations in different forms.

Thank you for your comments. We modified the expressions.

Therefore, to better capture complexity of CPSM, it is needed to develop an integrated index of CPSM based on the framework of CPSM for further investigating farmers’ role in enhancing crop-pollination service. (Line: 114-116)

**Issue 1. 8.:** L131-132: “fuzzy set quantitative comparative analysis” can be deleted, as the “fsQCA” was explained in L129.

Thank you for your advice. We deleted the “fuzzy set quantitative comparative analysis”.

Therefore, fsQCA is used to systematically examines the causes of different CPSM and the interactions between drivers thereby deepening understanding of configuration analysis of CPSM (Ragin & Strand 2008). (Line: 145-147)

**Issue 1. 9.:** L138-139: “crop-pollination service management” “CPSM”.

Thank you for your advice. We modified the expressions.

Recognizing the looming pollination crisis around the world, a handful of governments in developed countries have grappled with pollinator conservation and CPSM issues. (Line: 150-151)

**Issue 1. 10.:** L139-140: please add references for this example.

Thank you for your advice. We added references for this example.

For example, the European Union has proposed a series of management practices to promote pollinator conservation and enhance crop-pollination services (Boix-Fayos and de, 2023; Moldoveanu et al., 2024). (Line: 151-153)

Boix-Fayos, C., de Vente, J., 2023. Challenges and potential pathways towards sustainable agriculture within the European Green Deal. Agricultural Systems 207, 103634.

Moldoveanu, O.C., Maggioni, M., Dani, F.R., 2024. Environmental ameliorations and politics in support of pollinators. Experiences from Europe: A review. Journal of Environmental Management 362, 121219.

**Issue 1. 11.:** L147: delete the extra “Knapp”.

Thank you for your suggestions. We delete the “Knapp”.

Despite ecological research on the topic of pollinator conservation and pollination service management is abundant, there is few interdisciplinary research (Knapp et al., 2021). (Line: 158-160)

**Issue 1. 12.:** L148-149: the abbreviation ICSM refers to “integrated index of crop-pollination service management” in this objectives, is the “integrated” included in the abbreviations? Or it can be written as “integrated index of CPSM”.

Thank you for your advice. We revised the expression.

This study will fill the gap and its one objective is: (1) to propose an integrated index of CPSM based on the framework of CPSM. (Line: 160-161)

**Issue 2. 1.:** L157: add “of” after “framework”.

Thanks for your suggestions. We added “of” after “framework”.

A general framework of crop-pollination service management. (Line: 171)

**Issue 2. 2.:** L168: at least tomato and sunflower are not only relied on insect pollination, they also have the ability of self-pollination, it is inappropriate to use “complete dependence” for these plants. Maybe use partially dependence, and please add references for the plant examples.

Thank you very much. We revised the expression and added references for the plant examples.

The pollinator dependence of different crops differs greatly in extent which range from little or no dependence (e.g., wind-pollinated or self-pollinated cereals) to partially dependence (e.g., melon, tomato, and sunflower) and are intrinsically linked to plant breeding systems (Delgado-Carrillo et al., 2015., Shivanna, 2015). (Line: 181-184)

Delgado-Carrillo, O., Martén-Rodríguez, S., Ramírez-Mejía, D., Novais, S., Quevedo, A., Ghilardi, A., Sayago, R., Lopezaraiza-Mikel, M., Pérez-Trujillo, E., Quesada, M., 2024. Pollination services to crops of watermelon (Citrullus lanatus) and green tomato (Physalis ixocarpa) in the coastal region of Jalisco, Mexico. PLOS ONE 19, e0301402.

Shivanna, K.R., 2015. Management of Pollination Services to Enhance Crop Productivity, in: Bahadur, B., Venkat Rajam, M., Sahijram, L., Krishnamurthy, K.V. (Eds.), Plant Biology and Biotechnology: Volume I: Plant Diversity, Organization, Function and Improvement. Springer India, New Delhi, pp. 697-711.

**Issue 2. 3.:** L169: “plant breeding systems” may be a more suitable phrase instead of “plant physiology”.

Thanks for your suggestions. We modified the expression.

The pollinator dependence of different crops differs greatly in extent which range from little or no dependence (e.g., wind-pollinated or self-pollinated cereals) to complete dependence (e.g., melon, tomato, and sunflower) and are intrinsically linked to plant breeding systems (Delgado-Carrillo et al., 2015., Shivanna, 2015). (Line: 181-184)

**Issue 2. 4.:** L169: add “about” or “on” after “farmers’ observation”.

Thank you for your comment. We added “about” or “on” after “farmers’ observation”.

Contribution refers to farmers’ observations of the frequency of pollinator visits to crops (Fig. 1 Principle Ⅱ). (Line: 184-185)

**Issue 2. 5.:** L169-170: Please specify “the frequency of occurrence about pollinator’s visits”, does it mean that the relative abundance of different pollinator species, or specific pollination behaviour such as visitation rate, foraging speed, etc.

Thanks for your comment. It mean that the relative abundance of different pollinator species. And we revised the expression.

Contribution refers to farmers’ observations of the frequency of pollinator visits to crops (Fig. 1 Principle Ⅱ). (Line: 184-185)

**Issue 2. 6.:** L171: add “on” after “context-dependent”?

Thanks for your suggestion. We revised the expression.

The frequency of occurrence for pollinator’s visits to crops is often context-dependent on specific landscape conditions and management strategies. (Line: 185-187)

**Issue 2. 7.:** Fig.1: The figure itself should be self-explanatory, e.g., the specific meanings of A-F arrows should be indicated clearly. Appropriate terminologies or specific terms can be used here instead of using A-F. The limited contributions from the pollinators due to lack of pollinators or effective pollination behaviour may influence the sensitivity. However, if principle II refer to the pollinators’ visits to crops (L165-166), then the meaning of arrow C can be confusing when the supplementary pollination technique was added to the process. Please specify where this additional pollination technique located in the figure. Are there any other arrows that were not presented in the figure? For example, contribution may directly influence execution, farmers may adopt pollinator-related management before there were effects of yield and quality. When there were only a few bees available in the field, farmers may bring more bee hives before finishing of flowering.

Thank you for your valuable feedback. We understand your concern regarding the clarity of the arrows in Fig. 1 and have made the necessary revisions to enhance the figure’ self-explanatory nature. To improve clarity, we have labeled the arrows A-F to reflect the sequential influences in the CPSM framework. Each arrow represents the impact of one principle on another, and these links are now explained in detail in the revised framework.

Regarding your concern about Principle II and the inclusion of supplementary pollination techniques, we would like to clarify that this study focuses on natural pollination, and thus, the role of supplementary techniques, such as artificial pollination, is not considered within the scope of our research. We revised the framework to ensure that the principles reflect the role of natural pollination systems, and any potential confusion regarding supplementary pollination techniques has been addressed.

We believe these revisions provide greater clarity and more accurately reflect the relationships between the principles in the framework of CPSM. Thank you again for your insightful suggestions.

**Section 2. 1 A general framework of crop-pollination service management**

Here, the framework of CPSM is provided, which allows farmers to have greater flexibility in pollinator-supporting behaviors to adapt specific landscape conditions, crop varieties, and management strategies. The framework of CPSM, which outlines how farmers trust in the beneficial effects of pollinator-supporting behaviors based on pollination knowledge, linked with their involvement in pollinator-supporting practices, is presented to promote pollinator conservation and enhance crop-pollination service. The four underpinning principles of the framework are respectively dependence, contribution, sensitivity, and execution (Fig. 1). To better understand the framework of CPSM, the concepts of principles and the hypothesized links among them are explored below. Dependence represents farmers’ cognition of the pollination dependency of their crops (Fig. 1 Principle I). The pollinator dependence of different crops differs greatly in extent which range from little or no dependence (e.g., wind-pollinated or self-pollinated cereals) to partially dependence (e.g., melon, tomato, and sunflower) and are intrinsically linked to plant breeding systems (Delgado-Carrillo et al., 2015., Shivanna, 2015). Contribution refers to farmers’ observations of the frequency of pollinator visits to crops (Fig. 1 Principle Ⅱ). The frequency of occurrence for pollinator’s visits to crops is often context-dependent on specific landscape conditions and management strategies. Farmers’ observations on the contribution of pollinator-supporting behaviors are often essential to enhance farmers’ knowledge and insights about dependence (Fig. 1 arrow A). Sensitivity stands for farmers’ perceptions of the extent of yield or quality reduction in absence of pollinators compared to potential yield or quality (Fig. 1 Principle Ⅲ). In general, a higher dependency on crop-pollination service could improve farmers’ understanding of sensitivity in CPSM (Fig. 1 arrow B). Indeed, sensitivity might also be relevant to potential effects of contribution changes on farmers’ CPSM, and substituted technologies and other means (such as hand pollination) that farmers implemented in their fields (Fig. 1 arrow C). Execution indicates farmers’ adoption of agricultural practices that promote pollinator conservation and enhance crop-pollination service (pollinator-supporting practices) (Fig. 1 Principle Ⅳ). Farmers’ cognition of sensitivity is important for execution because it may affect individuals’ perception of the effectiveness of adaptive actions, allowing farmers to take diverse pollinator-supporting practices in different CPSM scenarios (Fig. 1 arrow D). The more diversified farmers’ adoption of pollinator-supporting practices, the greater their options for avoiding adverse effects caused by insufficient pollinator contributions. This approach also helps to optimize the discrepancies between farmers’ perceptions of pollinator dependence and those of researchers (Fig. 1 arrow E). At the same time, farmers’ willingness to adopt pollinator-supporting practices also is often limited by their knowledge of dependence (Fig. 1 arrow F). Here, the framework of CPSM highlights a positive, and often neglected, feedback loop of the relationship between farmers’ knowledge and experience and their pollinator-supporting behaviors (Fig. 1 the direction of blue and green arrows). The four principles (dependence, contribution, sensitivity, and execution) within the positive feedback loop of the framework of CPSM may reinforce each other, offering insights into how to implement on-farm CPSM that are more realistic and more suited to local agriculture landscapes. Because the framework of CPSM is built to explore how farmers manage crop-pollination service, it is necessary to assess on-farm CPSM among farmers by using the integrated index based on the framework of CPSM. The algorithm for the integrated index of CPSM is presented in Section 3.3. (Line: 172-212)

Fig. 1 A summarized general framework of CPSM. The boxes: four underpinning principles of the framework of CPSM; A-F arrows: hypothesized links among four principles (the direction of blue arrows represents increasing farmers’ trust in pollinator-supporting behaviors based on their pollination knowledge and experience; the direction of green arrows represents farmers’ involvement in pollinator-supporting practices to enrich their related knowledge and experience). For further explanation of principles and links see text.

**Issue 2. 8.:** L179: add “of” after “adoption”.

Thanks for your suggestion. We revised the expression.

Execution indicates farmers’ adoption of agricultural practices that promote pollinator conservation and enhance crop-pollination service (pollinator-supporting practices) (Fig. 1 Principle Ⅳ). (Line: 195-196)

**Issue 2. 9.:** L197: add “of the” after “complexity”.

Thanks for your comment. We revised the expression.

2.2 Theory of planned behavior and of the complexity theory (Line: 220)

**Issue 2. 10.:** L200-201: are there any references available for this statement?

Thanks for your comment. We added a reference for the statement.

The TPB was proposed by Ajzen and Fishbein (1977) on the basis of the Theory of Reasoned Behavior (TRB). (Line 222-223)

Ajzen, I. and M. Fishbein., 1977. "Attitude-Behavior Relations: A Theoretical Analysis and Review of Empirical Research." Psychol. Bull. 84: 888-918.

**Issue 2. 10.:** L204: government governmental

Thanks for your suggestions. We revised the expression.

Since there is no governmental policy targeting crop-pollination in Dengkou County, economic benefit remains the primary pursuit of local farmers when making decisions. (Line 244-246)

**Issue 2. 11.:** L205: please give a short description of Dengkou County, when it appears for the first time in the text.

Thank you for pointing this out. We added a brief description of Dengkou County when it is first mentioned in the text to provide context for readers.

Dengkou County, located in the Inner Mongolia Autonomous Region of China, is an agricultural area known for its production of crops such as sunflowers, melons, and wheat. Since there is no governmental policy targeting crop-pollination in Dengkou County, economic benefit remains the primary pursuit of local farmers when making decisions. (Line 243-246)

**Issue 2. 12.:** L208: what is “EI” stands for?

Thank you for your comment. EI stands for Economic Incentive and its full name is shown in the introduction section.

**Issue 2. 13.:** L278: add “a” after “used”.

Thank you for your suggestion. We added “a” after “used”.

We used a typical sampling approach to select 54 sample villages under sample towns and used a completely random sampling method to select 4 to 6 sample farmers from every target village. (Line 222-223)

**Issue 2. 14.:** L285-292: is this part belong to the Ethics Statement?

Thank you for your comment. It belongs to the Ethics Statement, which is a requirement of the journal.

**Issue 2. 15.:** L296-297: this part was repeated in L283-284.

Thank you for your comment. We deleted the repeated sentence.

**Issue 2. 16.:** L297-300: this part was repeated in L291-294.

Thanks for your advice. We deleted the repeated sentence.

**Issue 2. 17.:** L308: add “of” after “measurement”.

Thank you for your suggestion. We added “of” after “measurement”.

However, the measurement of farmers’ CPSM, which should attempt to consider specific regions and various crop types, is very difficult.

**Issue 2. 18.:** L321, 325, 329, 333: “scorce” “scores”.

Thanks for your advice. We revised the expression.

“*Knowledge_j_*” is scorces of *j*th element in dependence.

“*Observation_j_*” is scorces of *j*th element in contribution.

“*Perception_j_*” is scorces of *j*th element in senitivity.

“*Practice_j_*” is scorces of *j*th element in execution.

**Issue 2. 19.:** L327: column 7 and 8?

Thank you for your suggestion. We revised the expression.

*W_jⅡmax/min_* could be found in Appendix (Table 1, column 7 and 8).

**Issue 2. 20.:** L390: add references for the two software used.

Thank you for your comment. We added references for the two softwares used.

FsQCA3.0 and R4.1.1 software is used to conduct NCA-fsQCA (Huang et al., 2023). (Line 226-227)

Huang, Y., Bu, Y., Long, Z., 2023. Institutional environment and college students’ entrepreneurial willingness: A comparative study of Chinese provinces based on fsQCA. Journal of Innovation & Knowledge 8, 100307.

**Issue 2. 21.:** L393: why use Mann-Whitney U test here? It is used for nonparametric datasets. There are many tests to compare the differences for two groups. The reason for using this test is insufficient here. Please reconsider the selection of tests and specify the reasons for using the test.

Thank you for your comments. We have changed Mann-Whitney U test to independent sample t-test and modified the empirical results accordingly.

Independent sample t-test is an available tool to clarify the differences between two groups (Chatzi, 2025). To analyze configurations of CPSM more delicately, this study employs independent sample t-test to examine the differences between social-psychological factors and four principles of the framework of CPSM. (Line 230-233)

References:

Chatzi, A.V., 2025. Understanding the independent samples t test in nursing research. British journal of nursing (Mark Allen Publishing) 34, 56-62.

**Section 4. 5 CPSM’s principles analysis based on configuration**

To supplement the results of NCA-fsQCA further meticulously, independent sample T test is employed to examine whether there are differences in principles between high and low social-psychological factors, and results are shown in Table 7. Execution shows a difference between farmers with high and low AT. Farmers with high AT have higher implementation of CPSM. Execution and sensitivity show differences between farmers with high and low SN. Farmers with low SN in Dengkou county are more likely to appreciate the effects of CSPM on crop production, and are more willing to implement CPSM measures. Contribution, sensitivity, and execution show differences between farmers with high and low PBC. Farmers with high PBC have confidence in CSPM, place a greater emphasis on crop-production and have a higher level of practice. Dependence, contribution, sensitive, and execution show differences between farmers with high and low EI. Farmers with high EI have higher pollination trust and implement more CPSM. (Line 564-574)

**Table 7** CPSM’s principles analysis based on configuration (Independent sample T test).

| TPB | Principle | Mean ± standard deviation | | *t*-value | *p*-value |
| --- | --- | --- | --- | --- | --- |
|  |  | Low | High |  |  |
| AT | Execution | 5.12±0.69 | 5.27±0.64 | -1.88 | 0.061* |
|  | Dependence | 4.54±0.65 | 4.60±0.65 | -0.74 | 0.457 |
|  | Contribution | 5.31±0.66 | 5.41±0.57 | -1.35 | 0.178 |
|  | Sensitivity | 4.21±0.65 | 4.22±0.55 | -0.13 | 0.894 |
| SN | Execution | 5.31±0.72 | 5.11±0.57 | 2.41 | 0.016** |
|  | Dependence | 4.57±0.64 | 4.57±0.66 | 0.03 | 0.979 |
|  | Contribution | 5.41±0.66 | 5.32±0.53 | 1.23 | 0.219 |
|  | Sensitivity | 4.35±0.61 | 4.05±0.55 | 4.24 | 0.000*** |
| PBC | Execution | 4.98±0.73 | 5.27±0.64 | -2.85 | 0.005*** |
|  | Dependence | 4.51±0.67 | 4.59±0.64 | -0.70 | 0.486 |
|  | Contribution | 5.24±0.65 | 5.40±0.59 | -1.76 | 0.080* |
|  | Sensitivity | 3.99±0.61 | 4.27±0.58 | -3.11 | 0.002*** |
| EI | Execution | 5.15±0.68 | 5.38±0.62 | -2.53 | 0.012** |
|  | Dependence | 4.50±0.67 | 4.76±0.53 | -2.99 | 0.03** |
|  | Contribution | 5.42±0.67 | 5.26±0.41 | 1.94 | 0.054* |
|  | Sensitivity | 4.17±0.60 | 4.32±0.57 | -1.90 | 0.058* |

**Issue 4.1.:** The indications and citations should not be included in the Results part (e.g., L463, 471, section 4.4.4, etc.), they should be moved to the Discussions. Also, the reasons for using a specific analytical tool should be in the Methodology, please check if there is a repeated information between Methodology and Results.

Thank you for your valuable comments. We have carefully revised the manuscript according to your suggestions. We removed citations and indications from the Results section (e.g., L463, 471, section 4.4.4) and moved relevant discussions to the Method section, ensuring that the Results section strictly presents findings without interpretative elements. We reviewed the Methodology section to ensure that the rationale for selecting specific analytical tools is clearly stated there. Additionally, we checked for redundant information between the Methodology and Results sections and have revised them accordingly to improve clarity and avoid repetition.

As shown in Fig. 7, all conditions are below the consistency threshold of 0.9, indicating that no necessity conditions existed. (Line 507-508)

And two methods don’t generate contradictions and can complement each other. (Line 515)

This study selects to change the consistency threshold (adjusted from 0.8 to 0.85) to reprocess the sample data. The results indicate that configurations of high and low integrated index of CPSM are identical to the subset of original results, and the resulting configuration is consistent, thus, the conclusion is stable. (Line 559-562)

**Issue 4.2.:** L404: when present mean values in the text, standard deviations or standard errors should be added.

Thank you for your insightful suggestion. We have now added the corresponding standard deviations alongside the mean values in the text at L404 to ensure a more comprehensive presentation of the data. This revision enhances the clarity and accuracy of the reported results.

The mean and standard deviation of agricultural acreage are 4.79 hectares and 73.50, respectively. (Line 441-442)

**Table 1** Descriptive statistics of demographics

| Variable | Description | Min. | Mean | Max. | Std. Dev. |
| --- | --- | --- | --- | --- | --- |
| Gender | 0=males, 1=females | 0 | 0.33 | 1 | 0.47 |
| Education | 6= primary school, 9= junior high school, 12= senior high school, 15= junior college | 6 | 8.09 | 15 | 2.39 |
| Age | >0 year | 26 | 56.63 | 76 | 8.70 |
| Agricultural acreage | >0 hectare | 0.13 | 4.79 | 40.00 | 73.50 |
| EI | Agricultural profit ($) | 51.62 | 5293.50 | 76548.67 | 8236.26 |

**Issue 4.3.:** L405: the EI values were already presented in Table 1.

Thank you for your comments. We removed the EI value for line 405.

**Issue 4.4.:** L413: sensitive sensitivity

Thank you for your advice. We changed sensitive to sensitivity.

For principle Ⅲ (sensitivity), respondents believe that crop yield and quality are strongly influenced by CPSM. (Line 449-451)

**Issue 4.5.:** L414: executive execution

Thank you for your suggestion. We revised executive to execution.

For Principle IV (execution), respondents’ practices of installing beehives and avoiding pesticide spraying at flowering time are more highly implemented. (Line 451-452)

**Issue 4.6.:** L442: add a space between “ofTable2”.

Thank you for your suggestion. We added a space between “ofTable2”.

In model 6 (Column 7 of Table 2), all independent variables are included. (Line 485)

**Issue 4.7.:** Figure 6: the abbreviations shown in the figure and table should be explained in the legend. There is a lack of description and explanations of colored values of -1 to 1. Also, there is a lack of explanations of the values in the table and parenthesis, respectively.

Thank you for your comments and suggestions. Our abbreviations have all been shown previously, and a detailed explanation of the abbreviations within the diagram would lead to too much confusion. We added relevant image notes based on your suggestions.

Correlation matrix diagram of variables. From -1 (red) to 1 (blue) indicates from perfect negative correlation to perfect positive correlation. The data within the graph are the values represented by the colours.

The correlation coefficients between the integrated index of CPSM and Education, AT, PBC and lnEI are 0.16,0.2,0.12 and 0.21, respectively, indicating that the integrated index of CPSM is positively correlated with Education, AT, PBC and EI. The correlation coefficient between the integrated index of CPSM and SN is -0.17, indicating that the integrated index of CPSM and SN are negatively correlated. (Line 469-472)

Fig. 6. Correlation matrix diagram of variables. From -1 (red) to 1 (blue) indicates from perfect negative correlation to perfect positive correlation. The data within the graph are the values represented by the colors.

**Issue5.:** In general, there were many repeated information and descriptions in the discussions compared to the previous text, the authors are recommended to check throughout the text. The indications presented in the discussion should attach the relevant statistical results, and refer to a specific figure and table. Many of the statements and speculations in the discussion are lack of supporting references. The implications should also be extended to a broader scale, i.e., more references and/or comparisons of other studies at the national level or global levels are needed.

Thank you for your thorough review and valuable suggestions. We carefully addressed your comments and made revisions: (1) Reduced repetition in the discussion. We carefully reviewed the text and removed repetitive information in the discussion section. (2) Inclusion of statistical results. We incorporated relevant statistical results into the discussion and made clear references to specific figures and tables to support our statements. (3) Addition of supporting references. We addressed the lack of supporting references by adding citations to relevant studies throughout the discussion. (4) Broader implications with global comparisons: To extend the implications to a broader scale, we included comparisons with national and global-level studies. This includes examples from Europe, America, and other regions to highlight how the proposed policies and practices can be applied or adapted internationally, reinforcing the relevance of our findings beyond the local context.

The modified text is shown below: (Line 578-716)

**Section 5. Discussions**

Section 5. 1 Farmers’ demographics and CPSM

The descriptive statistics of the different principles of CPSM are shown in the Appendix (Table 3: Descriptive statistics of the different principles of CPSM), the small difference between the means of the different principles indicates that there is little difference between the values of the different principles. Therefore, it is more valuable to explore relationships between demographics and the integrated index of CPSM than to just compare scores of different principles. Fig. 5 shows that the larger farming scales, the higher integrated index of CPSM, i.e. agricultural acreage is positively correlated with the integrated index of CPSM. Contribution and dependence of CPSM can be more clearly perceived by farmers during cultivation (Raderschall et al., 2021). It may be that the larger area of crop cultivation, the more farmers attach importance to crop-pollination services, and therefore the easier it is to implement CPSM. However, excessive-scale farming tends to intensify agricultural production, and the resulting homogenization of agricultural landscapes will pose a serious threat to pollinator habitat management (Betts et al., 2019). This broadly supports farmers’ trust in the value of crop-pollination services, therefore, farmers with large planting areas can be guided to actively participate in CPSM to strengthen their efforts to implement pollinator-supporting practices, such as creating ‘gardens’ for bees at field boundaries (Jelsma et al., 2019). Fig. 6 shows that the correlation coefficient between education and the integrated index of CPSM is 0.19, indicating that the effect of education on the integrated index of CPSM is significantly positive, i.e. farmers with higher levels of education have higher integrated index. Well-educated farmers tend to have wider ranges of knowledge and are better able to understand CPSM (Zeweld et al., 2020). Farmers with higher education often have stronger self-learning abilities and problem-solving abilities and are more likely to accept new technologies about CPSM, which is conducive to farmers to better coping with challenges arising from enhancing CPSM. Therefore, improving farmers’ education level is an important strategy to enhance CPSM, and it is necessary to incorporate pollination knowledge into farmers’ education and production.

Section 5. 2 Configurations to enhance farmers’ CPSM

To achieve enhance CPSM, farmers can via different configurations: AT-PBC path (CPSM1), AT-EI path (CPSM2), and PBC-EI path (CPSM3). It is noteworthy that the absence of SN is observed in all groups with high integrated index of CPSM. The influence is reflected in that differences between dependence and contribution among farmers with high and low SN are not significant, and the sensitivity of farmers with high SN is lower than those with low SN. Farmers who are more affected by SN are less sensitive to the value of CPSM in regulating crop yield and quality. Therefore, SN does not support CPSM for farmers in Dengkou County (Hipólito et al., 2018). However, ignoring regional social context and simply reducing the role of SN on CPSM mean that a unified intervention strategy will not achieve the maximum efficiency of CPSM. CPSM is a kind of management with positive externalities, and external intervention should emphasize guiding farmers’ external environment by formulating relevant policies to produce SN with positive externalities on CPSM. Based on three configurations to achieve better CSPM, the intervention strategy should design different optimal CPSM schemes for farmers’ heterogeneity.

AT-PBC path (CPSM1). In CPSM, AT is indirectly reflected in farmers’ evaluation of crop-pollination. Farmers with high AT values have stronger cognitions of the regulatory value of pollinators on crop-production, which directly affects the action orientation of farmers’ CPSM (Knapp et al., 2021). In addition, high PBC not only provides confidence in executive for farmers’ pollinator protection, but also can carry psychological construction of achieving better CPSM, so that the implementation of CPSM has room for continuous strengthening and improvement (Westlake & Hunt, 2021). However, under conditions of high AT and high PBC, the absence of SN indicates that public service support has an irreplaceable role in promoting farmers to implement CPSM. For example, public information platforms such as “Agricultural Technology Promotion in China” not only provide real-time agricultural technology promotion but also publicize pollination knowledge (Gao et al., 2023). The social effect caused by public service not only directly creates SN with positive externality, but also indirectly improves individuals’ AT and PBC. Therefore, combination of high AT and high EI, with complete public service to support CPSM, constitutes a realization path of better CPSM.

AT-EI path (CPSM2). Farmers with high AT trust the value of crop-pollination services to agricultural production and ecosystems, so they are more likely to support the implementation of CPSM (Hall & Martins, 2020). Agricultural benefits are a key attribute of EI, farmers with high EI are more sensitive to the positive impact of CPSM on crop-production (Geslin et al., 2017). Further, for farmers with both high AT and EI, pollinator protection is not only an environmental action, but also an important management to increase agricultural production. Therefore, in terms of pollinator protection, high AT is the action orientation of high EI farmers, and high EI provides monetary gain for high AT farmers. However, in presence of high AT and EI, the absence of SN highlights the indispensable role of government support in promoting farmers to implement CPSM. For example, eco-compensation policies that provide positive externalities SN and economic support for pollinator protection can potentially improve farmers’ AT about CPSM (Brown et al., 2021). Therefore, combination of high AT and high EI, as well as effective pollination compensation policies, is an important way to enhance CPSM.

PBC-EI path (CPSM3). Farmers with high PBC tend to have stronger self-perceptions of practical ability and corresponding values of CPSM, which directly constitutes psychological construction about pollinator conservation trust (Ge et al., 2021). Farmers with high EI have stronger capital input potential and agricultural output efficiency, and generally have more resources and funds to bear costs of implementing CPSM. Further, farmers with high PBC and EI are also more likely to seek education and training related to CPSM and invest in advanced technologies and equipment that support CPSM, and confidently apply knowledge and technology to implement CPSM based on strong sense of self-efficacy. These investments and practices can enhance both pollinator conservation and agricultural output benefits, ultimately becoming an important driver of a virtuous cycle of pollinator conservation and agricultural benefits. However, in presence of high PBC and EI, the absence of SN suggests that efficient market supports play an integral role in promoting farmers to enhance CPSM. In 2022, the difference between per capita income and expenditure of permanent rural residents in Dengkou County is $1,748, whereas the difference with the minimum EI required for the optimal level of CPSM is $152.27^[1]^. Therefore, the disposable income of local farmers needs to be improved. Agricultural products premium about pollination can boost agricultural income and generate SN with positive externality for CPSM from market perspectives. Therefore, combination of high PBC and high EI, supported by effective market supports about pollination, is an important path to enhance CPSM.

Section 5. 3 Policy implications

Section 5.3.1 Motivating CPSM by enhancing farmers’ attitude about pollinator

In pursuit of pollinator protection in agriculture, government agencies can develop policies aimed at improving farmers’ AT by promoting education and fostering supportive pollination cultures. Results suggest that farmers’ willingness to adopt pollinator-friendly practices is influenced by education. So, governments worldwide should establish more agricultural education platforms (e.g., farmer field schools, science and technology academies) to improve farmers’ awareness and AT towards crop-pollination and pollination practices (Dai et al., 2024). The promotion of pollination practices should also be strengthened through official media and social platforms (e.g., new media of government affairs) to create a “social classroom” that complements education platform (Faxon, 2023). These recommendations are consistent with global practices, as agricultural education and public awareness campaigns have shown positive impacts in regions such as the European Union, where national governments have funded programs to promote pollinator conservation (Radojičić, 2024). Similar initiatives in Latin America and Africa have demonstrated the effectiveness of farmer training programs in boosting adoption of sustainable practices (Pretty et al., 2003).

Section 5.3.2 Innovating and Popularizing CPSM Technologies

Farmers’ mastery and application of advanced technology can enhance efficiency of implementing CPSM and increase their confidence and motivation to control pollination practices. The government should attach importance to the innovation capital investment of pollination technology, encourage technology-oriented enterprises to connect with farmers in order to solve practical technical problems faced by farmers in CPSM (Nunes, 2024). Focus on the research and development of CPSM’s new agricultural mechanization equipment (e.g., agricultural drone assisted pollination), and increase innovative pollinator protection technologies (e.g., smart beehives) (Hadjur et al., 2022). Establish information platforms related to pollinator protection to promote pollination technology, and guide farmers to use advanced CPSM technology through agricultural technology training (Barlow et al., 2020). Countries such as the U.S. and Canada have pioneered the integration of smart technologies into agriculture, leading to a significant increase in pollination efficiency and farmers’ engagement in pollinator protection strategies (Dicks et al, 2016). Similarly, China’s recent push for technological innovation in agriculture has led to successful pilot programs involving drone-assisted pollination (Xiong et al., 2024).

Section 5.3.3 Establishing “government + market” pollination compensation model to strengthen economic incentive

Results show that, in execution principle, installing beehives, keeping their own bees or buying insect pollination services are not well implemented by farmers in Dengkou County. Government should establish pollination protection laws to provide legal protection for crop-pollination services, and the key is to clarify compensation objects (e.g., farmers, beekeepers, merchants), compensation contents (e.g., beekeeping and purchase of pollination services), and how to compensate (e.g., direct subsidies, pollination transition and maintenance subsidies) (Hall and Steiner, 2019). The enforcement agencies responsible for pollination protection should be strengthened to ensure strict adherence to pollination protection laws, and coordination between national legislation and local departments should be improved to support effective implementation (Zhang et al., 2023). Different pollination compensation standards should be set in different regions. Pollination compensation standard in Dengkou County should be $152.27, which is the lowest EI ($1900.27) of the optimal CPSM minus the difference between farmers’ income and expenditure. Market-oriented, pollination compensation in market gradually transitions from price support to direct payment of consumer (Ruben, 2014). Insect-pollination certification can be provided to agricultural products that meet standards to increase consumer trust (Khachatryan et al., 2017). Farmers need to ensure the quality of insect-pollinated agricultural products and enhance consumers’ value recognition through marketing strategies (e.g., packaging and branding of insect-pollinated agricultural products) (Henríquez-Piskulich et al., 2021). Compared to existing policies for the protection of pollination services in the EU and the US, this policy implication allows for a more flexible and regionally targeted approach, tailored to local economic conditions and pollination needs, while ensuring a gradual shift from government-supported subsidies to market-driven incentives that have the potential to contribute to long-term sustainability and greater farmer participation (Porto et al., 2020).

Reference:

Barlow, S.E., O’Neill, M.A., 2020. Technological advances in field studies of pollinator ecology and the future of e-ecology. Current Opinion in Insect Science 38, 15-25.

Dai, Z., Wang, Q., Jiang, J., Lu, Y., 2024. Influence of university agricultural technology extension on efficient and sustainable agriculture. Scientific Reports 14, 4874.

Dicks, L.V., Viana, B., Bommarco, R., Brosi, B., Arizmendi, M.d.C., Cunningham, S.A., Galetto, L., Hill, R., Lopes, A.V., Pires, C., Taki, H., Potts, S.G., 2016. Ten policies for pollinators. Science 354, 975-976.

Faxon, H.O., 2023. Small farmers, big tech: agrarian commerce and knowledge on Myanmar Facebook. Agriculture and Human Values 40, 897-911.

Gao, Y., Wang, Q., Chen, C., Wang, L., Niu, Z., Yao, X., Yang, H., Kang, J., 2023. Promotion methods, social learning and environmentally friendly agricultural technology diffusion: A dynamic perspective. Ecological Indicators 154, 110724.

Hadjur, H., Ammar, D., Lefèvre, L., 2022. Toward an intelligent and efficient beehive: A survey of precision beekeeping systems and services. Computers and Electronics in Agriculture 192, 106604.

Hall, D.M., Steiner, R., 2019. Insect pollinator conservation policy innovations at subnational levels: Lessons for lawmakers. Environmental Science & Policy 93, 118-128.

Henríquez-Piskulich, P.A., Schapheer, C., Vereecken, N.J., Villagra, C., 2021. Agroecological Strategies to Safeguard Insect Pollinators in Biodiversity Hotspots: Chile as a Case Study, Sustainability.

Khachatryan, H., Rihn, A.L., Campbell, B., Yue, C., Hall, C., Behe, B., 2017. Visual Attention to Eco-Labels Predicts Consumer Preferences for Pollinator Friendly Plants, Sustainability.

Nunes, L.J.R., 2024. Exploring the present and future of biomass recovery units: technological innovation, policy incentives and economic challenges. Biofuels 15, 375-387.

Porto, R.G., de Almeida, R.F., Cruz-Neto, O., Tabarelli, M., Viana, B.F., Peres, C.A., Lopes, A.V., 2020. Pollination ecosystem services: A comprehensive review of economic values, research funding and policy actions. Food Security 12, 1425-1442.

Pretty, J.N., Morison, J.I.L., Hine, R.E., 2003. Reducing food poverty by increasing agricultural sustainability in developing countries. Agriculture, Ecosystems & Environment 95, 217-234.

Radojičić, N., 2024. The Clash of the Policies: The Joint Effect of EU Cohesion Policy and Common Agricultural Policy on the Public Support for European Integration. JCMS: Journal of Common Market Studies n/a.

Ruben, R., 2024. From market-based development to value chain transformation: What markets can (not) do for rural poverty alleviation? Journal of Rural Studies 109, 103328.

Xiong, C., Zhang, Y., Wang, W., 2024. An Evaluation Scheme Driven by Science and Technological Innovation—A Study on the Coupling and Coordination of the Agricultural Science and Technology Innovation-Economy-Ecology Complex System in the Yangtze River Basin of China, Agriculture.

Zhang, Z., Zhang, G., Hu, Y., Jiang, Y., Zhou, C., Ma, J., 2023. The evolutionary mechanism of haze collaborative governance: novel evidence from a tripartite evolutionary game model and a case study in China. Humanities and Social Sciences Communications 10, 69.

**Issue 5.1.:** L535-540: this part was repeated in the introduction L96-99. The section 5.1 is more like the reasons for choosing the analytical tools and can be moved to the methodology.

Thank you for your advice. We deleted L535-540 and moved the rest of section 5.1 to the Methods section.

Methodology:

We develop an integrated index of CPSM as a continuous variable only relative to itself. Another advantage of the integrated index is that each principle contains different specific survey questions, rather than a single question being set to represent multi-principles. In addition, the weight of elements in each principle is accounted for to prevent the creation of an unfair advantage for one principle over others. However, depending on local situation and the goal of enhancing CPSM, some principles of the integrated index may be more important than others. Therefore, to ensure that the integrated index fits ecological context in Dengkou County, we consulted regional pollinator experts and non-academic stakeholders in framework construction. The approach strengthens the social network of the integrated index. The integrated index’s algorithms based on the framework of CPSM can be generalized to other regions, but it is recommended to use similar methods to derive different elements and weights based on regional characteristics. (Line 389-397)

**Issue 5.2.:** L 552: please specify what “little difference” means by providing specific statistical results.

Thank you for your comments and suggestions. We provided specific statistical results to specify what “little difference” means.

The descriptive statistics of the different principles of CPSM are shown in the Appendix (Table 3: Descriptive statistics of the different principles of CPSM), the small difference between the means of the different principles indicates that there is little difference between the values of the different principles. (Line 579-582)

Appendix Table 3 Descriptive statistics of different principles of CPSM.

| Principle | Min. | Mean | Max. | Std. Dev. |
| --- | --- | --- | --- | --- |
| Contribution | 3.93 | 6.66 | 5.37 | 0.61 |
| Dependence | 2.93 | 6.00 | 4.58 | 0.65 |
| Sensitivity | 3.00 | 5.66 | 4.22 | 0.60 |
| Execution | 3.59 | 6.64 | 5.22 | 0.67 |

**Issue 5.3.:** L554-555: when present results in the discussion, the relevant statistical results should be presented as well, referring to the specific table or figure.

Thank you for your advice. We added figure references to the statistical results.

Fig. 5 shows that the larger farming scales, the higher integrated index of CPSM, i.e. agricultural acreage is positively correlated with the integrated index of CPSM. (Line 583-585)

**Issue 5.4.:** L564: specific statistical results should be presented here.

Thanks for your suggestion. We added figure references to the statistical results.

Fig. 6 shows that the correlation coefficient between education and the integrated index of CPSM is 0.19, indicating that the effect of education on the integrated index of CPSM is significantly positive, i.e. farmers with higher levels of education have higher integrated index of CPSM. (Line 593-596)

**Issue 5.5.:** L575-578: this part was mentioned previously.

Thanks for your comment. We deleted the duplicate parts.

**Issue 5.6.:** L583: which statement is this reference referred to?

Thanks for your comment. Our conclusion here is the research result of this paper and does not need to be supported by references. Therefore, we removed the misquoted references.

Farmers who are more affected by SN are less sensitive to the value of CPSM in regulating crop yield and quality. Therefore, SN does not support CPSM for farmers in Dengkou County. (Line 608-610)

**Issue 5.7.:** L590: the definition of AT was repeated.

Thanks for your suggestion. We removed the duplicate definition of AT.

**Issue 5.8.:** L599-601, 629-631, 666-668: please add references to.

Thanks for your advice. We added references to these statements and examples.

For example, public information platforms such as “Agricultural Technology Promotion in China” not only provide real-time agricultural technology promotion but also publicize pollination knowledge (Gao et al., 2023). (Line 625-627)

Reference:

Gao, Y., Wang, Q., Chen, C., Wang, L., Niu, Z., Yao, X., Yang, H., Kang, J., 2023. Promotion methods, social learning and environmentally friendly agricultural technology diffusion: A dynamic perspective. Ecological Indicators 154, 110724.

In 2022, the difference between per capita income and expenditure of permanent rural residents in Dengkou County is 1,748$, whereas the difference with the minimum EI required for the optimal level of CPSM is 152.27$. (Line 655-657)

Reference: Dengkou County 2023 government work report (<http://www.nmgdk.gov.cn/zfxxgkdk/fdzdgknrdk/dkgzbg/202302/t20230206_499330.html>).

Pollination compensation standard in Dengkou County should be 152.27$, which is the lowest EI ($1900.27) of the optimal CPSM minus the difference between farmers’ income and expenditure. (Line 704-706)

Reference: Dengkou County 2023 government work report (http://www.nmgdk.gov.cn/zfxxgkdk/fdzdgknrdk/dkgzbg/202302/t20230206_499330.html).

**Issue 5.9.:** Section 5.4: there is a lack of references in this sections, please add references for many of the examples in this section.

Thank you for your advice. We added references to the examples in Section 5.4.

Motivating CPSM by Enhancing Farmers’ AT about pollinator. In pursuit of pollinator protection in agriculture, government agencies can develop policies aimed at improving farmers’ AT by promoting education and fostering supportive pollination cultures. Results suggest that farmers’ willingness to adopt pollinator-friendly practices is influenced by education. So, governments should establish more agricultural education platforms (e.g., farmer field schools, science and technology academies) to improve farmers’ awareness and AT towards crop-pollination and pollination practices (Dai et al., 2024). The promotion of pollination practices should also be strengthened through official media and social platforms (e.g., new media of government affairs) to create a “social classroom” that complements education platform (Faxon, 2023). (Line 664-677)

Innovating and Popularizing CPSM Technologies. Farmers’ mastery and application of advanced technology can enhance efficiency of implementing CPSM and increase their confidence and motivation to control pollination practices. The government should attach importance to the innovation capital investment of pollination technology, encourage technology-oriented enterprises to connect with farmers in order to solve practical technical problems faced by farmers in CPSM (Nunes, 2024). Focus on the research and development of CPSM’s new agricultural mechanization equipment (e.g., agricultural drone assisted pollination), and increase innovative pollinator protection technologies (e.g., smart beehives) (Hadjur et al., 2022). Establish information platforms related to pollinator protection to promote pollination technology, and guide farmers to use advanced CPSM technology through agricultural technology training (Barlow et al., 2020). (Line 679-692)

Establishing “government + market” pollination compensation models to strengthen the role of EI in promoting CPSM. Results show that, in execution principle, installing beehives, keeping their own bees or buying insect pollination services are not well implemented by farmers in Dengkou County. Government should establish pollination protection laws to provide legal protection for crop-pollination services, and the key is to clarify compensation objects (e.g., farmers, beekeepers, merchants), compensation contents (e.g., beekeeping and purchase of pollination services), and how to compensate (e.g., direct subsidies, pollination transition and maintenance subsidies) (Hall and Steiner, 2019). The enforcement agencies responsible for pollination protection should be strengthened to ensure strict adherence to pollination protection laws, and coordination between national legislation and local departments should be improved to support effective implementation (Zhang et al., 2023). Different pollination compensation standards should be set in different regions. Pollination compensation standard in Dengkou County should be 152.27$, which is the lowest EI ($1900.27) of the optimal CPSM minus the difference between farmers’ income and expenditure. Market-oriented, pollination compensation in market gradually transitions from price support to direct payment of consumer (Ruben, 2014). Insect-pollination certification can be provided to agricultural products that meet standards to increase consumer trust (Khachatryan et al., 2017). Farmers need to ensure the quality of insect-pollinated agricultural products and enhance consumers’ value recognition through marketing strategies (e.g., packaging and branding of insect-pollinated agricultural products) (Henríquez-Piskulich et al., 2021). (Line 695-716)

Reference:

Dai, Z., Wang, Q., Jiang, J., Lu, Y., 2024. Influence of university agricultural technology extension on efficient and sustainable agriculture. Scientific Reports 14, 4874.

Faxon, H.O., 2023. Small farmers, big tech: agrarian commerce and knowledge on Myanmar Facebook. Agriculture and Human Values 40, 897-911.

Nunes, L.J.R., 2024. Exploring the present and future of biomass recovery units: technological innovation, policy incentives and economic challenges. Biofuels 15, 375-387.

Hadjur, H., Ammar, D., Lefèvre, L., 2022. Toward an intelligent and efficient beehive: A survey of precision beekeeping systems and services. Computers and Electronics in Agriculture 192, 106604.

Barlow, S.E., O’Neill, M.A., 2020. Technological advances in field studies of pollinator ecology and the future of e-ecology. Current Opinion in Insect Science 38, 15-25.

Hall, D.M., Steiner, R., 2019. Insect pollinator conservation policy innovations at subnational levels: Lessons for lawmakers. Environmental Science & Policy 93, 118-128.

Zhang, Z., Zhang, G., Hu, Y., Jiang, Y., Zhou, C., Ma, J., 2023. The evolutionary mechanism of haze collaborative governance: novel evidence from a tripartite evolutionary game model and a case study in China. Humanities and Social Sciences Communications 10, 69.

Ruben, R., 2024. From market-based development to value chain transformation: What markets can (not) do for rural poverty alleviation? Journal of Rural Studies 109, 103328.

Khachatryan, H., Rihn, A.L., Campbell, B., Yue, C., Hall, C., Behe, B., 2017. Visual Attention to Eco-Labels Predicts Consumer Preferences for Pollinator Friendly Plants, Sustainability.

Henríquez-Piskulich, P.A., Schapheer, C., Vereecken, N.J., Villagra, C., 2021. Agroecological Strategies to Safeguard Insect Pollinators in Biodiversity Hotspots: Chile as a Case Study, Sustainability.

# Response to reviewer 3:

**Issue 1:** However, the study does not address cultural backgrounds (including local traditions and religion), which are often pivotal in shaping farmers' perceptions, knowledge, and decisions about agricultural practices. Cultural values, traditions, and norms can significantly influence attitudes (AT), perceived behavioral control (PBC), and economic considerations, which are central to the TPB framework used in this research. The omission of cultural factors may limit the depth and applicability of the findings, especially given the rural context of Dengkou County, where traditional beliefs and community practices likely play a role in shaping agricultural behavior.

Explore cultural and regional factors that might influence the applicability of the findings to other settings beyond Dengkou County. To enhance the study, it would be valuable to integrate an analysis of cultural influences on CPSM behaviors. For instance, exploring traditional agricultural practices, beliefs about pollination, and local community norms could provide a richer understanding of farmers’ decision-making processes. Incorporating cultural variables into the survey or conducting qualitative interviews to examine these aspects would add depth and nuance to the findings. Such an approach would contextualize the results within the socio-cultural environment of Dengkou County, making the study more robust and actionable. By addressing the role of cultural background, the research could offer a more comprehensive perspective on the factors driving CPSM, ultimately enhancing its theoretical and practical contributions to sustainable agriculture.

Thank you for your thoughtful and valuable feedback. We appreciate your suggestion to explore the role of cultural factors in shaping farmers’ behaviours within the Theory of Planned Behaviour (TPB) framework. As you pointed out, cultural background, including local traditions, beliefs, and community norms, plays a significant role in influencing the core TPB variables such as attitudes, perceived behavioural control (PBC), and economic incentives.

We agree that cultural values, traditions, and social norms are crucial factors in shaping farmers’ decisions, especially in rural contexts like Dengkou County. Indeed, these cultural influences often manifest through subjective norms (SN), which capture the social pressures or expectations that individuals perceive within their communities. While subjective norms in the TPB framework inherently reflect cultural and social influences, we recognize that our study’s current focus may not sufficiently highlight how deeply ingrained cultural values and traditions directly affect farmers’ behaviours related to pollinator conservation.

But we lack a cultural database, which is beyond the scope of our project funding. Given the significance of these cultural factors, we believe that integrating them more explicitly into future research could provide richer insights into how cultural influences shape agricultural decision-making. Our current study, by focusing on the core TPB components, lays the groundwork for understanding the role of subjective norms, but we acknowledge that a more detailed exploration of cultural influences could deepen our understanding of farmers’ motivations and decision-making processes, particularly in the context of pollinator-supporting behaviours.

We appreciate your constructive feedback, and we will certainly consider expanding the exploration of cultural factors in future studies to fully capture the complex social dynamics that influence farmers’ pollination management practices.

Thank you again for your insightful suggestions.

**Issue 2:** While the study focuses on socio-economic and behavioral factors, it could benefit from integrating ecological data, such as the diversity and abundance of pollinators in the surveyed farms, to correlate management practices with actual pollination outcome. The study is well-structured and provides valuable insights into CPSM. By incorporating ecological factors, validating tools, and exploring regional differences, the analysis can become even more robust and impactful. These suggestions can enhance the study’s ability to inform effective CPSM strategies and policies.

Thank you for your thoughtful and constructive feedback. We appreciate your suggestion to incorporate ecological data, such as the diversity and abundance of pollinators, to better correlate management practices with actual pollination outcomes.

While we agree that integrating ecological factors could strengthen the study and provide a more comprehensive understanding of the relationship between CPSM practices and pollination effectiveness, we would like to clarify that our research primarily focuses on socio-economic and behavioural factors, which align with the scope of our project’s funding and objectives. Unfortunately, the collection of ecological data, such as detailed pollinator diversity or abundance, is beyond the scope of our current study and not within the resources allocated for this research.

We do recognize the value of incorporating ecological variables into CPSM studies to validate management practices and explore regional differences in pollinator populations. In future research, we plan to consider the integration of ecological factors as a key avenue for further investigation. We believe that combining socio-economic and ecological data will offer a more holistic view of pollination management and contribute to more effective, region-specific CPSM strategies and policies.

Thank you again for your valuable insights. We will take these suggestions into account for future studies and hope to build on this foundational research by incorporating ecological aspects in subsequent work.

**Issue 3:** A cross-sectional survey provides a snapshot, but a longitudinal approach could better capture changes in farmer behavior over time and the long-term impact of interventions. This may relooked at or justification for exclusion provided.

Thank you for your valuable feedback and insightful suggestion regarding the potential benefits of using a longitudinal approach. We understand that a longitudinal design could offer a deeper understanding of changes in farmer behaviour over time and the long-term impact of interventions.

However, we would like to clarify that our study relies on cross-sectional data, which we believe is well-suited for the specific research objectives and methods employed, particularly fuzzy-set qualitative comparative analysis (NCA-fsQCA). The cross-sectional design allows us to capture a snapshot of farmers’ attitudes, behaviours, and perceptions at a particular point in time, which aligns with the aim of our study—to understand the factors influencing current CPSM practices and to identify causal configurations that drive pollinator-supporting behaviours.

While we recognize the value of panel data for tracking changes over time, the constraints of our study, including data availability and project scope, made it difficult to implement a longitudinal approach. Given the focus of our research on understanding farmers’ current behaviours and their decision-making processes at a specific point in time, we believe that the cross-sectional data provides a solid foundation for the analysis conducted.

We agree that a longitudinal approach could provide richer insights into the long-term dynamics of CPSM adoption. This is something we plan to consider for future studies, particularly as we expand the scope of our research and explore the temporal evolution of pollinator management practices.

Thank you again for your thoughtful suggestion. We hope this clarification addresses your concern and demonstrates the appropriateness of the cross-sectional approach for this particular study.

**Issue 4:** The mention of consulting regional pollinator experts and non-academic stakeholders in the discussion section, rather than the methodology, is a notable issue. It creates a gap in the transparency of the research process. If these consultations played a role in constructing the framework, they should have been explicitly described in the methodology to clarify how these insights influenced the study's design.

To improve, the consultation process should be added to the methodology section, detailing how experts and stakeholders contributed to framework development. Additionally, the influence of these consultations on the research outcomes should be explicitly stated, ensuring the study reflects a comprehensive and well-informed approach to the ecological context.

I strongly recommend relocating the following section from the supplementary materials to the methods section, as it is essential to the study and directly supports the extensive deliberations presented in the discussion and also presented in data analysis:

"To ensure the elements are relevant for Dengkou County, we reviewed literature, conferred with experts, and performed test interviews with members of the survey population. To determine the weights associated with principles and elements, a variant of the Delphi method known as Mini-Delphi (estimate-talk-estimate) was employed (Pan et al., 1996). This approach facilitates a collective assessment of a predetermined set of questions by experts, allowing them to adjust their views through structured discussions. For this study, three experts with extensive knowledge of agricultural pollination in Dengkou County, specifically in pollination management, were consulted by the Dengkou County Agricultural Extension Service."

Including this section in the methods would provide much-needed clarity on how the principles and weights were derived, which is central to the study's framework. Furthermore, its prominent role in the discussion underscores its importance, making its inclusion in the methods section critical for readers to fully understand and contextualize the findings. This move would enhance the study’s transparency, methodological rigor, and alignment between the methods and discussion sections.

Thank you for your insightful suggestion. We agree that the consultations with regional pollinator experts and stakeholders should be explicitly included in the methodology section to enhance transparency. We revised the manuscript to move the description of the expert consultations, the Mini-Delphi method, and their influence on framework development from the supplementary materials to the methods section.

To ensure the elements are relevant for Dengkou County, we reviewed literature, conferred with experts, and performed test interviews with members of the survey population. To determine the weights associated with principles and elements, a variant of the Delphi method known as Mini-Delphi (estimate-talk-estimate) was employed (Pan et al., 1996). This approach facilitates a collective assessment of a predetermined set of questions by experts, allowing them to adjust their views through structured discussions. For this study, three experts with extensive knowledge of agricultural pollination in Dengkou County, specifically in pollination management, were consulted by the Dengkou County Agricultural Extension Service. (Line 371-378)

**Issue 5. Critique of placement in main text vs. supplementary material**

Relevance to core methodology. Equations like these are critical to understanding the methodology used to calculate scores and indices central to the study. They detail how the analysis is conducted and provide transparency, ensuring replicability. However, they are dense and mathematical, which might distract readers from the broader methodological narrative if presented in the main text.

Purpose of the equations. o If the equations are essential for readers to follow and evaluate the results and methodology, they should remain in the main text. o If they are primarily supporting details for replicability or validation purposes, they could be moved to supplementary material.

Accessibility for the audience. o For a broad audience, including practitioners and policymakers, presenting such detailed equations in the main text might reduce readability. o For a more specialized audience, such as academic researchers familiar with these methods, keeping them in the main text might be justified.

Current context. o The equations are highly detailed and seem to be supported by a reference to the appendix, where additional data and variables (e.g., weights, algorithms) are described. This redundancy suggests they could be summarized in the main text with a clear reference to supplementary material for those seeking further detail.

Recommendation: balanced approach

• Main Text: Provide a concise summary of the methodology, outlining the key steps in plain language. Include one representative equation (e.g., Eq. 1) to illustrate the process, but avoid including all equations.

• Supplementary Material: Move the detailed equations (Eqs. 2-6) and algorithmic descriptions to the supplementary material, as they are technical details that support the core methodology but are not critical for the broader understanding of the study.

Suggested rewrite for main text. In the main text, you could say:

"To calculate the scores for each principle, we summed the maximum points of the elements within each principle and computed the corresponding scores based on weighted sums of the selected elements (Eq. 1). This process involved calculating respondents' scores for dependence, contribution, sensitivity, and execution using weighted principles and elements (detailed in Supplementary Material, Eqs. 2-6). These scores were then aggregated to obtain the Primary ICSM and subsequently converted to ICSM scores ranging from 0 to 100 (see Supplementary Material for the complete algorithm)."

Thank you for your suggested changes. We have revised the text and appendices according to the balance you suggested. Below is the revised version of the main text and appendix incorporating your suggested changes:

**Main text:**

To calculate the scores for each principle, we summed the maximum points of the elements within each principle and computed the corresponding scores based on weighted sums of the selected elements (Eq. 1). This process involved calculating respondents' scores for dependence, contribution, sensitivity, and execution using weighted principles and elements (Appendix: Algorithm for principles’ scores). These scores were then aggregated to obtain the primary integrated index and subsequently converted to the integrated index’s scores ranging from 0 to 100 (Appendix：Algorithm for converting the *Primary*_*ICSM* into *ICSM*)). (Line 353-359)

**Appendix:**

**Algorithm for principles’ scores**

Corresponding principles’ scores are calculated for each principle based on the weighted sum of the selected elements. As shown in Eq. 2,3,4,5.

${\text{P}\text{S}}_{\text{Ⅰ}}\text{=}\sum_{\text{j=}\text{1}}^{\text{4}} \frac{\text{(}\text{Knowledge}_{\text{j}}\text{*}\text{W}_{\text{j}\text{Ⅰ}\text{max}}\text{)}}{\text{Max score of principle}_{\text{Ⅰ}}}\text{+}\frac{\text{(}\text{(}\text{1}\text{-}\text{Knowledge}_{\text{j}}\text{)}\text{*}\text{W}_{\text{j}\text{Ⅰ}\text{min}}\text{)}}{\text{Max score of principle}_{\text{Ⅰ}}}$	(2)

*PS_Ⅰ_* is respondents’ scores of dependence, “*Knowledge_j_*” is scorces of *j*th element in dependence. *W_jⅠmax/min_* is max or min weight set for *j*th element in dependence. *W_jⅠmax/min_* can be found in Appendix (Table 1, column 7 and 8).

${\text{P}\text{S}}_{\text{Ⅱ}}\text{=}\sum_{\text{j=}\text{1}}^{\text{4}} \frac{\text{(}\text{Observation}_{\text{j}}\text{*}\text{W}_{\text{j}\text{Ⅱ}\text{max}}\text{)}}{\text{Max score of principle}_{\text{Ⅱ}}}\text{+}\frac{\text{(}\text{(}\text{1}\text{-}\text{Observation}_{\text{j}}\text{)}\text{*}\text{W}_{\text{j}\text{Ⅱ}\text{min}}\text{)}}{\text{Max score of principle}_{\text{Ⅱ}}}$	(3)

*PS_Ⅱ_* is respondents’ scores of contribution, “*Observation_j_*” is scorces of *j*th element in contribution. *W_jⅡmax/min_* is max or min weight set for *j*th element in contribution. *W_jⅡmax/min_* could be found in Appendix (Table 1, column 7 and 8).

${\text{P}\text{S}}_{\text{Ⅲ}}\text{=}\sum_{\text{j=}\text{1}}^{\text{2}} \frac{\text{(}\text{Perception}_{\text{j}}\text{*}\text{W}_{\text{j}\text{Ⅲ}\text{max}}\text{)}}{\text{Max score of principle}_{\text{Ⅲ}}}\text{+}\frac{\text{(}\text{(}\text{1}\text{-}\text{Perception}_{\text{j}}\text{)}\text{*}\text{W}_{\text{j}\text{Ⅲ}\text{min}}\text{)}}{\text{Max score of principle}_{\text{Ⅲ}}}$	(4)

PS*_Ⅲ_* is respondents’ scores of sensitivity, “*Perception_j_*” is scorces of *j*th element in senitivity. *W_jⅢmax/min_* is max or min weight set for *j*th element in sensitivity. *W_jⅢmax/min_* could be found in Appendix (Table 1, column 7 and 8).

${\text{P}\text{S}}_{\text{Ⅳ}}\text{=}\sum_{\text{j=}\text{1}}^{\text{5}} \frac{\text{(}\text{Practice}_{\text{j}}\text{*}\text{W}_{\text{j}\text{Ⅳ}\text{max}}\text{)}}{\text{Max score of principle}_{\text{Ⅳ}}}\text{+}\frac{\text{(}\text{(}\text{1}\text{-}\text{Practice}_{\text{j}}\text{)}\text{*}\text{W}_{\text{j}\text{Ⅳ}\text{min}}\text{)}}{\text{Max score of principle}_{\text{Ⅳ}}}$	(5)

PS*_Ⅳ_* is respondents’ scores of execution, “*Practice_j_*” is scorces of *j*th element in execution. *W_jⅣmax/min_* is max or min weight set for *j*th element in execution. *W_jⅣmax/min_* could be found in Appendix (Table 1, column 7 and 8).

Thirdly, each principle’s score is weighted and summed to obtain primary integrated index. Calculation formula is shown as Eq. 6.

$\text{Primary\_ICSM}\text{=}\sum_{\text{i}\text{=Ⅰ}}^{\text{Ⅳ}} \text{PS}_{\text{i}}\text{*}\text{W}_{\text{i}}$	(6)

*Primary_ICSM* is respondents’ primary integrated index’s scores. *W_i_* indicates weight of *PS_i_*, which could be found in Appendix (Table 1, column 3).

**Algorithm for converting the primary_ICSM into ICSM**

*ICSM* are transformed from *primary_ICSM* to the range of 0-100. Max score of *primary*_*ICSM* is the sum of the weights of principles, which is 25.333. Min score of *primary*_*ICSM* is the sum of the minimal scores of principles, which is 12.999. Algorithm for converting the *primary_ICSM into ICSM* is shown in Eq. 1:

*ICSM*=100**Primary_ICSM* / (25.333-12.999)-12.999 / (25.333-12.999)	(1)

Weights of principles and minimal scores of principles could be found in Appendix (Table 1, column 3 and 8)

**Issue 6:** The authors introduced the Theory of Planned Behavior (TPB) and its extension using economic incentives (EI) as a framework for analyzing crop-pollination service management (CPSM). However, their explanation lacks a thorough introduction and context for readers unfamiliar with TPB. The explanation of the Theory of Planned Behavior (TPB) in the manuscript is inadequate, lacking a thorough introduction to its core concepts. Key components like attitude (AT), subjective norm (SN), and perceived behavioral control (PBC) are briefly mentioned without sufficient context or real-world examples, making it inaccessible to readers unfamiliar with the theory. Additionally, the manuscript introduces complexity theory in the section title but fails to elaborate on it, causing confusion. The inclusion of economic incentives (EI) to extend TPB is not adequately justified, as assumptions about farmers’ motivations lack supporting evidence. The section's structure is disorganized, moving abruptly between concepts without a clear link. To improve, the authors should explain TPB in detail, justify its relevance to crop-pollination management, and integrate economic incentives with supporting data.

Thank you for your insightful feedback. We revised the section to provide a more detailed explanation of TPB’s core concepts, such as attitude (AT), subjective norm (SN), and perceived behavioral control (PBC), along with relevant real-world examples to enhance clarity. Regarding the integration of economic incentives (EI) with TPB, we have elaborated on how EI influences farmers’ behavioral intentions, specifically in relation to CPSM. We have also added supporting evidence from previous studies to justify the relevance of EI in motivating farmers’ adoption of pollinator-supporting practices. We have clarified the complexity theory, ensuring a more coherent structure and logical flow between the concepts. These revisions aim to make the theoretical framework more accessible and better aligned with the study’s focus.

**Section 2.2 Theory of planned behavior and of the complexity theory** (Line 353-398)

The TPB, a typical socio-psychological construct, was applied as the basic theoretical framework in analyzing farmers’ CPSM. The TPB was proposed by Ajzen and Fishbein (1977) on the basis of the Theory of Reasoned Behavior (TRB). Five components are included in the TPB: attitude, subjective norm, perceived behavioral control, behavioral intention and actual behavior. Attitude is derived from behavioral beliefs (a positive or negative evaluation of the outcome), Subjective norm originates from normative beliefs (social forces from others or social institutions), and perceived behavioral control is closely related to control beliefs (the internal factors that promote or hinder behavior) (Ajzen et al., 1986). Behavioral intention is defined as the anticipation of certain actions, which is collectively determined by the attitude, subjective norm and perceived behavioral control. According to the TPB, behavioral intention is the most critical predictor of actual behavior (Quine and Rubin, 1997). In addition, there may be interaction between attitude, subjective norm and perceived behavioral control (Ajzen, 1992). Meanwhile, the three components can be also used to predict actual behavior together with behavioral intention (Kautonen et al, 2013). TPB is highly relevant to CPSM as it helps explain how farmers' perceptions and behaviors regarding pollinator conservation are influenced by their attitudes, subjective norms, and perceived behavioral control. In the context of CPSM, the TPB framework enables the identification of key factors that shape farmers' intentions to adopt pollinator-supporting practices. Understanding these influences can help design more effective strategies to promote pollinator conservation and improve crop-pollination services at the farm level.

Previous studies have demonstrated that incorporating external incentives into TPB can better reveal special behavior (Daxini et al.,2019; Wang et al., 2019). However, the selection of indicators to extend TPB in a specific study largely depends on the characteristics of subject and goals. Dengkou County, located in the Inner Mongolia Autonomous Region of China, is an agricultural area known for its production of crops such as sunflowers, melons, and wheat. Since there is no governmental policy targeting crop-pollination in Dengkou County, economic benefit remains the primary pursuit of local farmers when making decisions. And economic efficiency plays a crucial role in implementing policies related to CPSM (Kleftodimos et al., 2021). In fact, as an external variable, economic factors have been used to optimize the TPB framework (Bravo-Monroy et al., 2015). Therefore, this study adds EI to TPB to provide a new perspective for extended TPB.

AT is defined as individuals’ subjective evaluations of performing specific behaviors, which is a crucial variable in predicting behaviors (Ajzen & Fishbein, 1977; Bohner & Dickel, 2011). In case of agricultural management, farmers’ adoption of agricultural practices will increase if they have positive AT toward action (Yang et al., 2022). Since the farmers’ behavior can be significantly affected and identified by their attitude, H1 is proposed:

H1: Farmers’ attitude (AT) has a positive relationship with their CPSM behavior.

SN represents individuals’ perceptions of pressures or expectations from external groups and affairs (Manning, 2009). These pressures or expectations arise from psychological conflict to perform target behaviors or not. Typically, SN resulting from positive external influences enhances individual behavior accordingly (Van et al., 2023). However, their intention to perform behaviors may decrease if behavior lacking policy guidance or contrary to social conventions (Hansson et al., 2012; Wan et al., 2017). SN not only directly reduces individual behaviors, but also reduces positive effects of AT and self-efficacy on actual behaviors (Fu et al., 2010). So, H2 is proposed:

H2: The subjective norm (SN) from society has a negative relationship with farmers’ degree of the implementation of CPSM.

PBC refers to the perception of one’s ability to control target behaviors (Ajzen & Madden, 1986). The stronger individuals’ perceptions of control over target behaviors, the more behavioral intentions will arise (Hagger et al., 2022). In scenarios of agricultural practice, PBC accounts for the largest proportion of actual behavioral variance (Bagheri & Teymouri, 2022). Thus, as farmers’ beliefs about their control over CPSM increase, their related actions will also increase. So, H3 is proposed:

H3: Farmer’s perceived behavioral control (PBC) has a positive relationship with the extent to which they implement CPSM.

EI reflects the influence of economic agents to engage in specific behaviors due to monetarily rewarded (Hahn & Stavins 1992). Economic Incentive provides individuals with the money needed for the behavior, which directly motivates the individual to enhance that behavior (Aznar-Sanchez et al., 2021). As an agricultural operating practice characterized by improving ecological environment, the higher economic efficiency of CPSM, the higher farmers’ level of CPSM (Li et al, 2021). So, H4 is proposed:

H4：Higher EI positively supports higher farmers’ CPSM levels.

Complexity theory assumes that small random events (through positive feedback effects of increasing returns) can dynamically lead to multi-equilibria (MAY, 1972; Arthur, 1999). That is, complexity theory indicates that relationships between variables can be non-linear, and variables can produce different results in specific circumstances (for example, combinations of farmers’ socio-psychological factors of different intensities may have impacts on their CPSM decisions leading to many forms of management decisions) (Cantele et al., 2023; Hidalgo, 2021). Specifically, management elements vary among subjects (farmers), and it is up to the subjects to explore, react and continuously change their actions and strategies in response to different outcome (Arthur, 2021). Since agricultural management is a complicated socio-ecological system, enhancing and maintaining CPSM will require identifying multidimensional solutions instead of a single solution. This supports the formulation of differentiated schemes to enhance CPSM levels of different farmers based on different crop planting backgrounds and differentiated farmer characteristics. Therefore, different factors may form various CPSM solutions through complex combinations (Fig. 2). Consequently, H5 and H6 are proposed:

H5: CPSM can be influenced by mutually complementary or alternative effects consisting of AT, SN, PBC, and EI.

H6: Better CPSM may or may not exist under same conditions across different configurations, depending on the specific manner in which farmers’ Attitude (AT), Social Norms (SN), Perceived Behavioral Control (PBC), and Economic Incentive are integrated and balanced.

**Issue 7: The manuscript is littered with many sentences which require rewording to bring clarity. I have listed some below and follow up is required on others:**

**Issue 7. 1.:** Line 169: The sentence “Contribution refers to farmers’ observation the frequency of occurrence about pollinator’s visits to crops” is not well-worded and needs clarification for proper grammatical structure and meaning. Here's a suggested revision: “Contribution refers to farmers’ observations of the frequency of pollinator visits to crops”

Thank you for your helpful suggestion to improve the clarity and grammatical structure of the sentence. Below is the revised version based on your recommendation:

Contribution refers to farmers’ observations of the frequency of pollinator visits to crops. (Line 184-185)

**Issue 7. 2.:** Line 54: The sentence “Moreover, pollinator-dependent crops show higher agricultural expansion rates than other crops” is not well-worded and needs clarification for proper grammatical structure and meaning. Here's a suggested revision: “Suggestion: Additionally, crops that depend on pollinators exhibit higher rates of agricultural expansion compared to non-pollinator-dependent crops”

Thank you for your helpful suggestion to improve the clarity and grammatical structure of the sentence. Your proposed revision enhances readability and precision. Below is the revised version based on your recommendation:

Additionally, crops that depend on pollinators exhibit higher rates of agricultural expansion compared to non-pollinator-dependent crops. (Line 54-55)

**Issue 7. 3.:** Line 86: The phrase "it to a large extent has higher potential" is awkward and not grammatically sound. It needs rephrasing for clarity and flow

Thank you for pointing out the issue with the phrase. Below is the revised version of the sentence:

Since the framework of CPSM is a comprehensive framework with a view of multi-dimensional realities, measuring farmers’ CPSM based on the framework will be important and challenging (Roquer-Beni et al., 2021). (Line 105-108)

**Issue 7. 4.:** Line 183: The sentence “The more diversified are farmers’ adoption pollinator-supporting practices, the more options they must avoid adverse effects induced by inadequate contribution, optimizing discrepancies between farmers’ cognition of pollinator dependence and researchers” needs rewording. Suggested re-wording: “The more diversified farmers’ adoption of pollinator-supporting practices, the greater their options for avoiding adverse effects caused by insufficient pollinator contributions. This approach also helps to optimize the discrepancies between farmers’ perceptions of pollinator dependence and those of researchers”

Thank you for your thoughtful suggestion to improve the clarity and structure of the sentence. Below is the revised version based on your recommendation:

The more diversified farmers’ adoption of pollinator-supporting practices, the greater their options for avoiding adverse effects caused by insufficient pollinator contributions. This approach also helps to optimize the discrepancies between farmers’ perceptions of pollinator dependence and those of researchers. (Line 199-201)

**Issue 7. 5.:** Line 268: The sentence “Among these crops, sunflower and melon are more rely on pollination mediated by insects” needs rewording. Suggested rewording “ Among these crops, sunflower and melon rely more on pollination mediated by insects.”

Thank you for your helpful suggestion to improve the wording of the sentence. Below is the revised version based on your recommendation:

Among these crops, sunflower and melon rely more on pollination mediated by insects. (Line 311)

**Issue 7. 6.:** Line 283: The sentence “The start and end dates of the recruitment period for this study are 01/07/2021 and 31/08/2021, respectively.” is clear but could be improved for readability and formality. Suggested revision: "The recruitment period for this study began on July 1, 2021, and ended on August 31, 2021."

Thank you for pointing out that the date format is required by the journal and editor, and cannot be modified. I appreciate your understanding. In that case, I will retain the original date format while incorporating your suggestion to improve readability and formality. Here is the revised version:

The recruitment period for this study started on 01/07/2021 and concluded on 31/08/2021. (Line 326-327)

**Issue 7. 7.:** Line 76-81 To avoid repetitive use of 'on the other hand in two sentences following each other immediately, consider this ["While differences in perceptions between farmers and researchers or policymakers highlight an understanding and communication gap (Batie et al., 2009), these disparities also reflect variations in knowledge and background among local farmers. This is because the benefits of pollinator-supporting practices on yield can vary depending on management factors, and farmer behavior—an essential component of agricultural practices—is directly influenced by their local knowledge and experience (Batie et al., 2019)]

Thank you for your thoughtful suggestion to improve the flow and avoid repetition in the text. Below is the revised version based on your recommendation:

While differences in perceptions between farmers and researchers or policymakers highlight an understanding and communication gap (Batie et al., 2009), these disparities also reflect variations in knowledge and background among local farmers. This is because the benefits of pollinator-supporting practices on yield can vary depending on management factors, and farmers’ behavior—an essential component of agricultural practices—is directly influenced by their local knowledge and experience (Batie et al., 2019). (Line 93-98)

**Issue 7. 8.:** Line 308 : The sentence "However, the measurement farmers’ CPSM, which should attempt to consider specific regions and various crop types, is very difficult" is not clear. The sentence structure could be simplified for better readability. A more polished revision would be: "However, measuring farmers' CPSM, which should account for specific regions and various crop types, is challenging."

Thank you for your valuable feedback and for suggesting a clearer and more polished revision. Below is the revised version based on your recommendation:

However, measuring farmers’ CPSM, which should account for specific regions and various crop types, is challenging. (Line 349-350)

**Issue 7. 9.:** Line 663: The sentence “The enforcement agencies of pollination compensation will be refined to ensure the strict enforcement of pollination protection laws, and the central legislation 664 will be coordinated with the implementation of local departments” needs refinement. Suggested revision "The enforcement agencies responsible for pollination protection should be strengthened to ensure strict adherence to pollination protection laws, and coordination between national legislation and local departments should be improved to support effective implementation."

Thank you for your insightful suggestion to improve the clarity and precision of the sentence. Below is the revised version of the sentence based on your recommendation:

The enforcement agencies responsible for pollination protection should be strengthened to ensure strict adherence to pollination protection laws, and coordination between national legislation and local departments should be improved to support effective implementation. (Line 700-703)

**Issue 7. 10.:** Line 583: [Farmers more affected by SN are less sensitive to the value of CPSM 582 in regulating crop yield and quality, therefore, SN does not support CPSM for farmers in Dengkou County (Hipólito et al., 2018).] Suggestion revision "Farmers who are more affected by SN are less sensitive to the value of CPSM in regulating crop yield and quality. Therefore, SN does not support CPSM for farmers in Dengkou County (Hipólito et al., 2018)."

Thank you for your thoughtful suggestion to improve the clarity and readability of the sentence. Below is the revised version of the sentence based on your recommendation:

Farmers who are more affected by SN are less sensitive to the value of CPSM in regulating crop yield and quality. Therefore, SN does not support CPSM for farmers in Dengkou County. (Line 608-610)

**Issue 8:** Line 269: In the methodology. The sentence "unwritten regulations" may cause confusion, as it suggests rules that are not formally documented, which can be ambiguous in academic writing. To clarify, it’s better to use "informal regulations" if referring to practices that are followed but not codified. Additionally, the sentence uses "enough" twice, which makes it sound repetitive. To improve readability and flow, varying the language would help. The contraction "don’t" should also be avoided in formal writing, replaced with "do not" for a more professional tone. Lastly, the sentence structure could be refined to ensure greater clarity and formality. A revised version would be: "However, policies and informal regulations in Dengkou County do not give sufficient attention to crop-pollination services, and farmers lack adequate awareness of CPSM.

Thank you for your suggestions for improving the clarity and formality of the sentence. Below is the revised version of the sentence based on your recommendations:

However, policies and informal regulations in Dengkou County do not give sufficient attention to crop-pollination services, and farmers lack adequate awareness of CPSM. (Line 314-315)

**Issue 9:** Line 290: The passage provides important ethical information but lacks clarity and coherence. The phrase "this study did not involve human or animal experimentation" is unnecessary since it contradicts earlier mentions of human participants. It would also benefit from clearer explanation about how informed consent was obtained and how respondents' data was protected. Suggested revision: "The Ethics Committee of the School of Economics and Management, Inner Mongolia Normal University, Hohhot, China, approved this study. All procedures involving human participants adhered to ethical standards, including the Declaration of Helsinki. Informed consent was obtained from all participants, and data were collected securely via online software to protect personal information."

Thank you for your suggestions. We have revised the passage as suggested to ensure it is clear, coherent. The revised section now reads:

The Ethics Committee of the School of Economics and Management, Inner Mongolia Normal University, Hohhot, China, approved this study. All procedures involving human participants adhered to ethical standards, including the Declaration of Helsinki. Informed consent was obtained from all participants, and data were collected securely via online software to protect personal information. (Line 329-335)

**Issue 10:** The phrase "267 questionnaires are returned" should be rephrased to improve readability, and the connection between the questionnaires and other data sources should be more clearly established. Suggested revision: "A total of 267 completed questionnaires were returned, all of which were deemed valid. The questionnaire covered elements related to CPSM and TPB, with specific questions listed in the Appendix (Table 1: Weights of the principles and chosen elements used in ICSM; Table 2: Descriptive statistics of the elements used to measure TPB constructs).

Thank you for your insightful suggestions. The sentence “267 questionnaires are returned” was revised in accordance with the recommendations:

A total of 267 completed questionnaires were returned, all of which were deemed valid. The questionnaire covered elements related to CPSM and TPB, with specific questions listed in the Appendix (Table 1: Weights of the principles and chosen elements used in the integrated index of CPSM; Table 2: Descriptive statistics of the elements used to measure TPB constructs). (Line 342-345)

**Issue 11:** Placement of dollar sign not consistent. The dollar sign in the sentence is not correctly placed. It should come before the number for consistency with standard formatting. Example below [Pollination compensation standard in Dengkou County should be 152.27$, which is the lowest EI ($1900.27) of the optimal CPSM minus the difference between farmers’ income and expenditure.]

We would like to express our gratitude for highlighting the discrepancy in the placement of the dollar sign in our manuscript. We have conducted a thorough review of all instances where monetary values are mentioned, and we have rectified the placement of the dollar sign to ensure it appears before the number, in accordance with standard formatting conventions. Specifically, the sentence in question has been revised as follows:

Pollination compensation standard in Dengkou County should be $152.27, which is the lowest EI ($1900.27) of the optimal CPSM minus the difference between farmers' income and expenditure. (Line 704-706)

**Issue 12:** Check to see if the following were meant to be or sentences. • Motivate CPSM by improving farmers’ AT about pollinator. • Innovate and popularize technology about CPSM.

Thank you for your comments. We acknowledge that these headings were intended to encapsulate the core concepts of each section in a concise manner. To address the concerns raised and enhance clarity, we made the necessary revisions.

The first point has been modified to “Motivating CPSM by Enhancing Farmers’ AT about pollinator,”

and the second to “Innovating and Popularizing CPSM Technologies.” (Line 663 and 678)

1. Reference: Dengkou County 2023 government work report (http://www.nmgdk.gov.cn/zfxxgkdk/fdzdgknrdk/dkgzbg/202302/t20230206_499330.html).
